# Integrated single-cell and bulk transcriptome analysis of R-loop score-based signature with regard to immune microenvironment, lipid metabolism and prognosis in HCC

**DOI:** 10.3389/fimmu.2024.1487372

**Published:** 2025-01-09

**Authors:** Long Chen, Houzhi Yang, Xianfu Wei, Jianchao Liu, Xiuxin Han, Chao Zhang, Yongheng Liu, Yan Zhang, Yao Xu, Yiqin Li, Guowen Wang, Jinyan Feng

**Affiliations:** ^1^ National Key Laboratory of Draggability Evaluation and Systematic Translational Medicine, Tianjin’s Clinical Research Center for Cancer, Department of Bone and Soft Tissue Tumors, Tianjin Medical University Cancer Institute and Hospital, National Clinical Research Center for Cancer, Tianjin, China; ^2^ Department of Orthopedics, Qilu Hospital of Shandong University, Jinan, Shandong, China

**Keywords:** R-loop, single-cell RNA-sequencing, HCC, tumor immune microenvironment, lipid metabolism reprogram, CLTC

## Abstract

**Background:**

Hepatocellular carcinoma (HCC) is one of the most prevalent causes of cancer-related morbidity and mortality worldwide. Late-stage detection and the complex molecular mechanisms driving tumor progression contribute significantly to its poor prognosis. Dysregulated R-loops, three-stranded nucleic acid structures associated with genome instability, play a key role in the malignant characteristics of various tumors. However, the detailed role and mechanism of R-loops in HCC progression remain elusive and require further exploration. This study aimed to construct an R-loop scoring signature centered on prognosis and lipid metabolism, thereby enhancing our understanding of HCC progression and identifying potential therapeutic targets.

**Methods:**

In this study, we utilized the single-cell RNA-sequencing (scRNA-seq) data from HCC patients (GSE149614 and CRA002308) to construct an R-loop scoring model based on the identified R-loop regulator genes (RLRGs) related to HBV infection through WGCNA analysis. We also explored the tumor microenvironment and intercellular communication related to R-loop score. Additionally, a prognostic risk model based on the fatty acid metabolism-associated RLRGs was constructed using data from the TCGA database, and its association with immune infiltration, mutations, and drug sensitivity was analyzed. *In vitro* and *in vivo* experiments were performed to investigate the role of RLRG CLTC in lipid metabolism and HCC progression.

**Results:**

Using scRNA-seq data from HCC, we established an R-loop scoring model based on identified RLRGs related to HBV infection. Moreover, the more suppressive tumor immune microenvironment and stronger intercellular communication were displayed in malignant cells with high R-loop scores. The cell trajectory and cellular metabolism analysis exhibited a significant association between lipid metabolism and RLRGs. Additionally, we constructed a prognostic risk model consisting of 8 RLRGs related to fatty acid metabolism, which effectively evaluated the prognostic value, status of tumor immune microenvironment, gene mutations, and chemotherapeutic drug sensitivity for HCC patients. Notably, validation experiments suggested that CLTC could regulate lipid metabolism through R-loop formation and facilitate tumor progression in HCC.

**Conclusion:**

Collectively, our study proposes an R-loop scoring model associated with tumor immune microenvironment, lipid metabolism and prognostic value. CLTC, an R-loop regulator, emerges as a promising prognostic biomarker and therapeutic target, offering new insights into potential treatment strategies for HCC patients.

## Introduction

Hepatocellular carcinoma (HCC) ranks as the sixth most prevalent cancer and the third leading cause of cancer-related mortality globally, with approximately 758,000 fatalities and 865,000 new diagnoses annually (1). In addition to other risk factors such as aflatoxin exposure, heavy alcohol consumption, obesity, and type 2 diabetes, HBV or HCV chronic infection accounts for 21% to 55% of HCC worldwide (2). Recently, metabolic reprogramming, particularly dysregulation of lipid metabolism, has gained increasing attention as a critical factor in HCC pathogenesis, significantly influencing its initiation and progression (3–5). Current therapeutic approaches for HCC, including surgical resection, liver transplantation, and systemic therapies, are often hampered by late-stage diagnosis and high recurrence rates (6, 7). Therefore, further research is imperative to unravel the molecular mechanisms driving HCC and identify more effective diagnostic and therapeutic targets.

R-loops are dynamic, triple-stranded nucleic acid structures consisting of an RNA-DNA hybrid and a displaced single-stranded DNA that form during transcription (8). Physiological R-loops are involved in the regulation of RNA transcription and processing, epigenetic modification, DNA repair, and mitochondrial replication (8, 9). However, unscheduled R-loops can lead to DNA replication stalling and potential genomic instability, which requires cells to precisely maintain R-loop homeostasis by R-loop regulator genes (RLRG), including BRCA2, DHX9, PARP1, and RNASEH1 (10–12). Given that R-loops dysregulation can lead to DNA damage and cellular stress, extensive research has demonstrated their involvement in a variety of physiological processes, including immune regulation, metabolic reprogramming, and cellular communication (13, 14). Excessive accumulation of R-loops has been reported to activate the cGAS-STING inflammatory pathway, increasing the number of hematopoietic stem and progenitor cells (HSPCs), which are crucial for maintaining immune system homeostasis (15). Crossley MP and colleagues dedicated that R-loops accumulation could activate the innate immune response and regulate cytokine-mediated inflammatory cascades by inducing the reorganization of immune cell interactions, potentially contributing to diseases such as cancer (16).

In recent years, the role of aberrant R-loops in driving the malignant progression of various cancer types has received considerable attention. Zhang et al. were the first to construct an R-loop scoring model according to the identified RLRGs, comprehensively uncovered the underlying molecular mechanism of metabolic reprogramming and T cell exhaustion under R-loop score patterns, and revealed that low R-loop scores displayed glycolysis and epithelial-mesenchymal transition pathway activation and immune escape promotion, ultimately impairing antitumor therapeutic efficacy. Furthermore, inactivation of BRCA2 leads to the formation of abnormal R-loops, which subsequently induce DNA damage and chromosomal aberrations, potentially contributing to an increased risk of breast cancer (17). Besides, knockdown of THOC1 increases R-loop formation, leading to DNA damage, which reduces HCC cell proliferation and enhances cisplatin sensitivity (18). MTA2 promotes HCC stemness via enhancing R-loop formation, which transcriptionally suppresses BDH1 through forming a complex with HDAC2/CHD4 (19). However, further comprehensive studies are required to elucidate the precise roles and molecular mechanisms of R-loops in HCC.

By utilizing single-cell RNA sequencing (scRNA-seq) data derived from HCC, this study constructed an R-loop scoring model, uncovering a significant correlation between the tumor malignancy and HBV infection with the R-loop scores. Subsequently, we analyzed the association between R-loop scores and both the tumor immune microenvironment and intercellular communication. Furthermore, we identified that RLRGs were intricately linked to lipid metabolism, based on which we constructed an innovative prognostic model by incorporating bulk RNA-seq data. Notably, we discovered that CLTC, a crucial RLRG, was significantly upregulated in HCC, modulated R-loop-mediated lipid metabolism, and facilitated tumor progression. In conclusion, our findings provide a theoretical foundation for focusing on R-loops or RLRGs as novel prognostic and therapeutic targets for HCC.

## Materials and methods

### Data collection

The scRNA-seq data GSE149614 for HCC patients was acquired from GEO (https://www.ncbi.nlm.nih.gov/geo), and CRA002308 was acquired from GSA (https://ngdc.cncb.ac.cn/gsa). HCC bulk RNA-seq data were downloaded from TCGA (https://portal.gdc.cancer.gov/), ICGC (https://dcc.icgc.org), and GEO databases, respectively. The scRNA-seq data GSE149614 includes 8 pairs of primary liver tumor and non-tumor liver tissue samples, while CRA002308 comprises 7 pairs of liver tumor and normal tissue samples. The clinical information related to these tissue samples can be obtained from the supplementary materials of the corresponding published articles (20, 21). TCGA-LIHC samples with complete clinical information were utilized as the model training set, ICGC-LIRI-JP samples with complete clinical information were utilized as the model validation set, and HCC samples from the GEO database (accession number GSE76427) were utilized as the external validation set. The R-loop-related gene set was obtained from the R-loopBase database (https://rloopbase.nju.edu.cn/download.jsp), which included a total of 1,268 R-loop regulators.

### scRNA-seq data analysis and identification of the cell type

The HCC scRNA-seq data were systematically processed using the Seurat (v4.1.1) R package. First, a Seurat object was created using the CreateSeuratObject function, with the min.cells parameter set to 3 to exclude genes expressed in fewer than three cells. Subsequently, further filtering of the cell data was performed, which involved removing cells with fewer than 200 or more than 8000 detected genes, as well as cells exhibiting a mitochondrial gene proportion exceeding 20% or a hemoglobin gene proportion exceeding 5%. To minimize the impact of doublets, the doubletFinder v3 function from the DoubletFinder package was used to identify and filter potential doublets. Key parameters were set as PCs = 1:20 and pN = 0.25, meaning that 20 principal components were considered to estimate the probability of each cell being classified as a doublet at 0.25. The filtered data were subsequently normalized using the LogNormalize method, wherein the raw counts were scaled to a total gene expression of 10,000 per cell. Subsequently, 2,000 highly variable genes were identified using the FindVariableFeatures function, followed by normalization with the ScaleData function to mitigate the impact of technical noise. Dimensionality reduction was then performed using the RunPCA function, with the first 20 principal components selected for subsequent analyses. For batch effect correction in multi-sample data, the RunHarmony function of the Harmony package was used for data integration. The samples were treated as the grouping variable (group.by.vars = “sample”), with the integration strength parameter set to lambda = 1 and the clustering penalty parameter set to theta = 2. The Harmony method, based on Principal Component Analysis (PCA), corrects for batch effects by embedding and iteratively removing systematic biases specific to each dataset, enabling effective integration of cells from different samples so that they cluster together. Subsequently, UMAP dimensionality reduction was performed using the ‘umap-learn’ algorithm in the RunUMAP function to facilitate subsequent visualisation of the integrated data. After batch effect correction, the FindNeighbours function was used to calculate the distances between cells and construct a Shared Nearest Neighbour (SNN) graph. Cell clustering was then performed using the FindClusters function using the Louvain algorithm with a resolution parameter set to 0.3 to identify cell subpopulations. Finally, during the cell annotation phase, the automated annotations generated by the SingleR software were combined with known cell marker genes and manual corrections were made to further refine the annotations.

### Weighted gene coexpression network analysis on scRNA-seq data to uncover gene network modules

We grouped cell populations based on similar origins through random, non-overlapping sampling from the same tissue source and cell type, with each group containing 30 cells. The R package WGCNA (v1.71) was used to construct the co-expression network of R-loop regulators, with an optimal beta value of 4 selected as the minimum soft-thresholding power. The parameter minModuleSize was set to 30 for the dynamic tree cut function. Highly similar modules were identified by clustering and subsequently merged using a height cutoff of 0.25. Using the “Eigengenes” function, we found that the co-expressed gene modules were associated with several clinical features, and modules significantly correlated with HBV infection were selected.

### Building an R-loop scoring model with R-loop regulators

The R-loop score was calculated using the cal_CRDscore function from the R package “CRDscore” (v0.1.0). Briefly, the average gene expression levels were first calculated across all cells. Subsequently, a random R-loop score (S_random) was generated using a random sampling strategy. Next, the score for central expression data for each sample or cell (S_center) was calculated. Finally, the R-loop score was obtained by subtracting S_random from S_center. In bulk samples, the R-loop score was calculated based on the expression of prognosis-associated differential expression R-loop regulators. Based on the median R-loop score, cells or samples were categorized into high- and low-score groups.

### The risk-score model’s development and validation

We first conducted an analysis to investigate the correlation between 165 R-loop modeling genes and the fatty acid metabolism pathway and found that PYM1, CTPS1, CLTC, VDAC3, SRPK1, and NOSIP were positively correlated with fatty acid metabolism, while FANCG, YWHAZ, and SLC25A6 exhibited a negative correlation. In total, 9 genes were identified as being associated with the fatty acid metabolism pathway. Next, using logistic regression with the Least Absolute Shrinkage and Selection Operator (LASSO) for analysis, we selected the optimal model genes and identified 8 key genes. The risk score for each patient, designed to assess the prognostic value of the risk model, was calculated based on the risk coefficients of the model genes obtained through multivariable Cox regression analysis, along with the expression levels of these model genes. The specific formula used for calculation is as follows: Riskscore = (PYM1 exp) * 0.1635 + (CLTC exp) * -0.1623 + (VDAC3 exp) * 0.1596 + (SRPK1 exp) * 0.1631 + (NOSIP exp) * 0.0929 + (FANCG exp) * -0.0018 + (YWHAZ exp) * 0.0802 + (SLC25A6 exp) * -0.3306. Using this method, we assigned each patient a numerical score reflecting their prognostic risk based on the expression levels of the model genes. The ‘surv_cutpoint’ function was used to determine the optimal cut-off value, which was then used to stratify patients into high- and low-risk groups, with the results visualized using Kaplan-Meier curves. The same evaluation process was applied to an external validation cohort to further verify the effectiveness of the risk model.

### Cell lines

LO2, Hep3B, Huh-7, HCC-LM3, and HepG2 cells were obtained from the Cell Bank of the Chinese Academy of Sciences (Shanghai, China). Cells were cultured in Dulbecco modified Eagle medium (DMEM; Cellmax, Beijing, China) supplemented with 10% fetal bovine serum (Cellmax, Beijing, China) and 1% penicillin-streptomycin (Biosharp, Anhui, China) in a humidified incubator with 5% CO2 at 37°C.

### DRIP-qPCR

DRIP assays were performed as previously described (22). DNA was extracted from HCC-LM3 and HepG2 cells, followed by overnight incubation at 37°C in ecRNH buffer with or without treatment with ecRNH (Beyotime, Shanghai, China). RNA-DNA hybrids from the digested nucleic acids, either treated or untreated with ecRNH, were immunoprecipitated using the S9.6 antibody (Abcam, USA) and protein A/G beads (MCE, China) at 4°C overnight in IP buffer. The beads were then washed four times with IP buffer for 10 minutes at room temperature, and nucleic acids were eluted with elution buffer at 55°C for 1 hour. The immunoprecipitated DNA was analyzed by qPCR.

### R-loop dot blot assay

DNA samples were loaded onto an Amersham Hybond-N+ membrane (Solarbio, China). The membrane was UV crosslinked for 5 minutes and washed with PBST. After blocking with 5% non-fat milk, the membrane was incubated with the S9.6 antibody (1:1000, Abcam) overnight at 4°C. Dot blots were then incubated with HRP-conjugated anti-mouse IgG for 1 hour and visualized using an imaging system (Bio-Rad, USA).

### *In vivo* mouse models

Female BALB/c nude mice (4-5 weeks old) were obtained from SPF Biotechnology Co., Ltd. (Beijing, China) and maintained under specific pathogen-free (SPF) conditions at the Tianjin Medical University Cancer Institute and Hospital. A total of 1 × 10^7 HepG2 cells, transduced to express either sh-CLTC or sh-NC, were suspended in 200 µL of PBS and injected subcutaneously into the right flank of the mice. Tumor volumes were measured using a vernier caliper every five days and calculated using the formula: V = (length × width²)/2. After 28 days post-injection, the mice were euthanized, and the tumors were harvested, photographed, and weighed. The expression of CLTC, FASN, SCD, and Ki67 in the tumors was analyzed by immunohistochemistry. All animal experiments were conducted in compliance with standard operating procedures and ethical guidelines for animal welfare, with approval from the Animal Care Committee of the Tianjin Medical University Cancer Institute and Hospital.

### Statistical analysis

The differences in continuous variables between two groups were assessed using the independent samples Mann-Whitney U test. The Kruskal-Wallis test was applied to the differences between three groups. The Chi-square test was used to assess the differences of categorical variables between two groups. The statistical analyses in this study were conducted using R software version 4.0.5 and GraphPad Prism 9.5. Data are presented as mean ± standard deviation, and each experiment was independently repeated at least three times. The *P* value < 0.05 was considered statistically significant.

## Results

### Single-cell transcriptome profiles of HCC tumor and adjacent non-tumor samples

**Figure 1:**
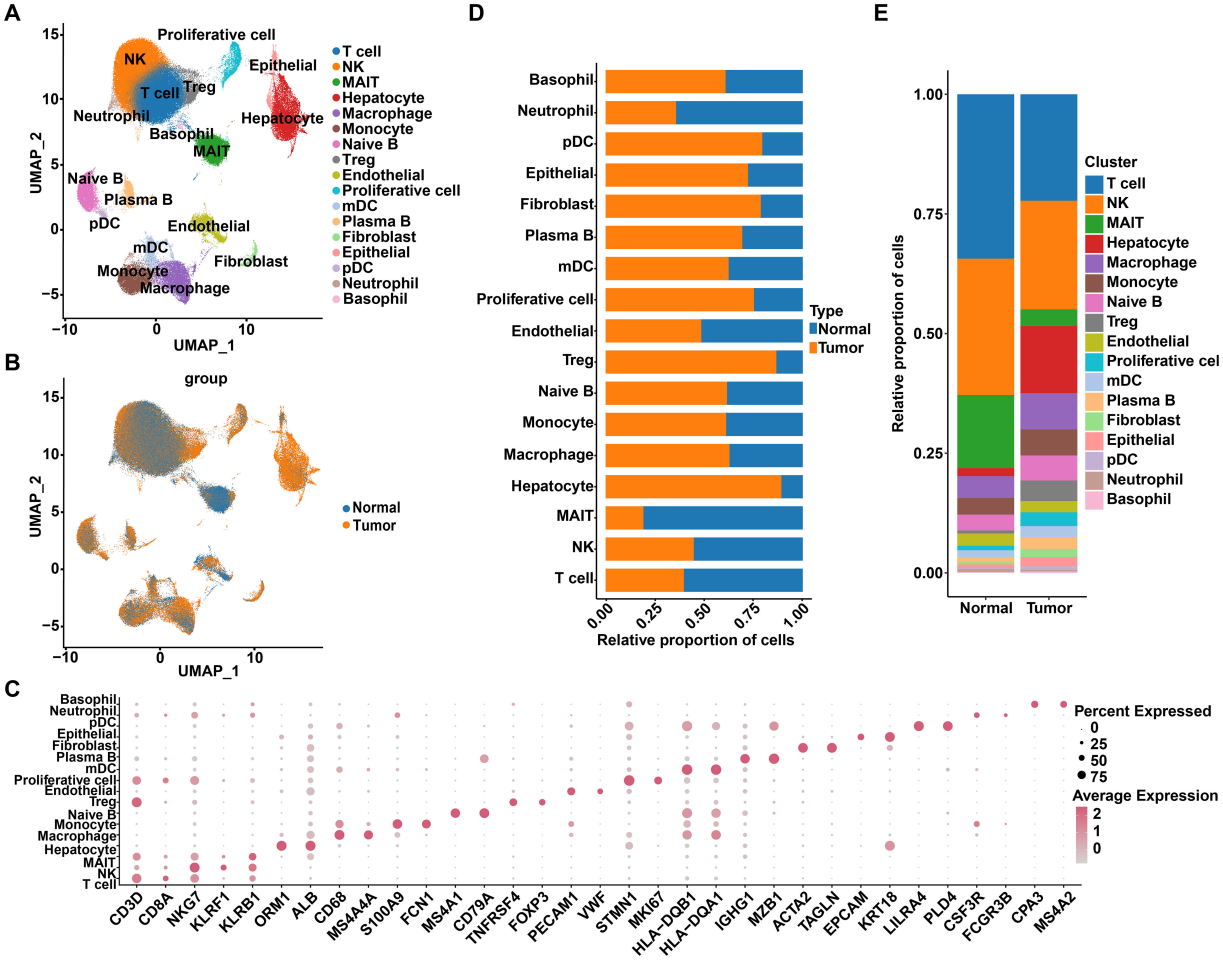
Single-cell transcriptome profiles of HCC tumor and adjacent non-tumor samples. **(A)** Uniform Manifold Approximation and Projection (UMAP) displays a total of 174,966 cells, divided into 17 cell types. **(B)** UMAP projection of HCC tissues and adjacent normal tissues. **(C)** Dot plot shows the expression of marker genes across different cell types. **(D)** The distribution of various cell types in tumor and normal tissues. **(E)** The proportion of each cell type in tumor and normal tissues.

To elucidate the association between R-loops and the tumor immune microenvironment landscape in HCC, we first performed an integrated data analysis of 15 liver cancer samples and their paired non-cancer samples, derived from the GSE149614 and CRA002308 datasets. The detailed clinical information of 15 HCC samples was shown in **Supplementary Figure S1A**, among which 9 displayed HBV infection, 3 were infected with HCV, and 3 were without viral infection. Following quality filtering, a total of 174,966 cells were detected, with a median cell count of 3,658 per sample and a median of 1,464 genes per cell, of which 93,193 and 81,773 cells originated from cancer samples and adjacent non-cancer samples, respectively. Using UMAP analysis for clustering, we identified 17 distinct cell subpopulations based on typical cell markers, including T cells, NK cells, MAIT cells, hepatocytes, macrophages, and monocytes (**Figures 1A–C**). Specifically, CD3D was used as a marker gene for T cells, while NKG7 indicated NK cells; ORM1 and ALB were markers for hepatocytes, and KRT18 defined epithelial cells (**Figure 1C**). Subsequently, comparing cell proportions between the two groups revealed that the proportions of immune cells, such as T cells and NK cells, were relatively high in both normal and tumor samples, whereas the proportions of hepatocytes, epithelial cells, and regulatory T cells (Treg cells) were higher in tumor samples compared to normal samples, indicating differential cell distribution (**Figures 1D, E**). To distinguish malignant from non-malignant populations, we assessed copy number variations (CNVs) in hepatocytes and epithelial cells, using plasma B cells as a reference. Among the 14,499 hepatocytes and 2,414 epithelial cells analyzed, 15,361 cells with high CNV scores were classified as malignant, while 1,552 cells with low CNV scores were identified as non-malignant, indicating that the majority of hepatocytes and epithelial cells were malignant (**Supplementary Figure S1B**).

### The R-loop score is elevated in HCC and is associated with advanced clinical stages in HCC patients

**Figure 2:**
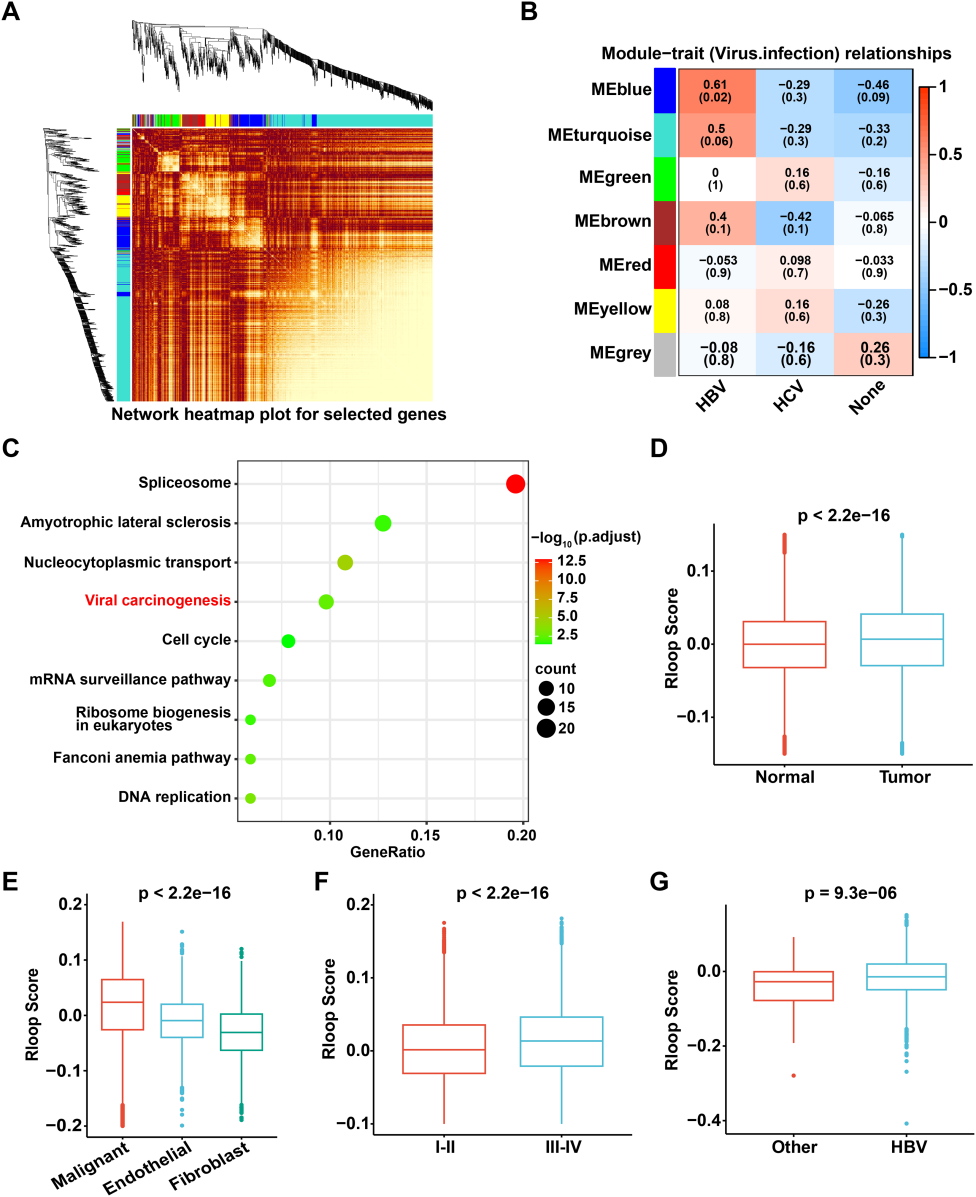
The R-loop score is elevated in HCC and is associated with advanced clinical stages in HCC patients. **(A)** WGCNA illustrates the associations among 1,268 R-loop regulator genes, with the color intensity representing the strength of the interactions. **(B)** Correlation analysis of gene co-expression modules with viral infection status. **(C)** KEGG pathway enrichment analysis of 165 genes from the blue module. **(D)** Comparison of R-loop scores between tumor and normal tissue (Wilcoxon rank-sum test). **(E)** Comparison of R-loop scores among different cell types (Kruskal-Wallis test). **(F)** Comparison of R-loop scores across different clinical stages (Wilcoxon rank-sum test). **(G)** Comparison of R-loop scores under different viral infection conditions (Wilcoxon rank-sum test).

To construct a gene co-expression network for HCC patients, we performed WGCNA and identified seven distinct modules related to TNM stage and viral infections based on the expression patterns of 1,268 RLRGs (23)(**Figures 2A, B**, **Supplementary Figure S2A**). Notably, the blue module demonstrated a significant positive correlation with HBV infection (**Figure 2B**). Considering that HBV infection is known to be closely associated with the development of HCC, we further selected the module genes associated with HBV infection (blue module), of which a total of 165 RLRGs were obtained. Functional enrichment analysis revealed that these genes were significantly enriched in pathways associated with viral carcinogenesis (**Figure 2C**, **Supplementary Figure S2B**). Additionally, a scoring model was constructed to evaluate R-loop levels using the 165 RLRGs identified. The R-loop scores of tumor samples were found to be significantly higher than those of normal samples (**Figure 2D**). Furthermore, compared to endothelial cells and fibroblasts, malignant cells had higher R-loop scores (**Figure 2E**). Stromal cells also had higher R-loop scores compared to immune cells (**Supplementary Figure S2C**). Meanwhile, elevated R-loop scores were associated with advanced clinical stages (**Figure 2F**). Noteworthy, patients with HBV infection exhibited significantly higher R-loop scores compared to non-infected patients (**Figure 2G**). The above results confirm that R-loops are remarkably elevated in HCC and may play crucial roles in tumorigenesis and disease progression.

### R-loop score describes characteristics of tumor immune microenvironment

**Figure 3:**
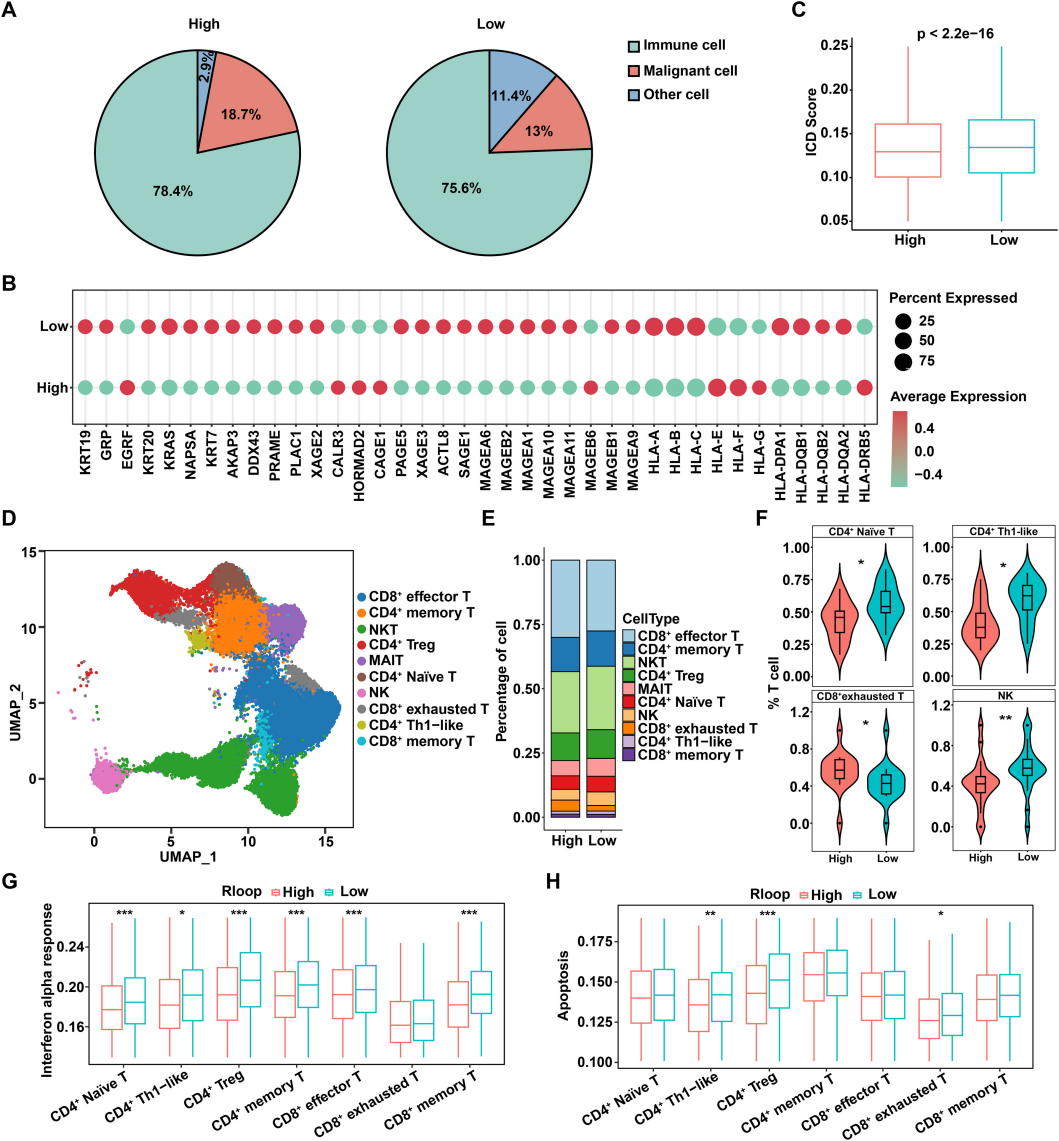
R-loop score describes characteristics of tumor immune microenvironment. **(A)** Comparison of the proportions of immune cells, malignant cells, and other cells across different R-loop score groups. **(B)** Expression levels of immune escape molecules in different R-loop score groups. **(C)** Differences in ICD scores among different R-loop score groups (Wilcoxon rank-sum test). **(D)** UMAP of T cells. NKT: natural killer T cells; CD4^+^ Treg: CD4^+^ regulatory T cells; MAIT: mucosal-associated invariant T cells; NK: natural killer cells; CD4^+^ Th1-like: T helper type 1-like CD4^+^ T cells. **(E)** Proportions of T-cell subsets between high and low R-loop score groups. **(F)** Comparison of the proportions of T-cell subpopulations with significant differences between high and low R-loop score groups (Wilcoxon rank sum test). **(G)** Comparisons of IFN-α response scores for T-cell subsets between high and low R-loop score groups (Wilcoxon rank-sum test). **(H)** Comparisons of apoptosis scores for T-cell subsets between high and low R-loop score groups (Wilcoxon rank-sum test). *P < 0.05; **P < 0.01; ***P < 0.001, *P* < 0.05 was considered statistically significant.

Considering that tumor immune microenvironment poses a significant barrier to cancer treatment and promotes tumor progression (24), we further investigated the relationship between R-loops and immune status. Based on the above R-loop scoring model, tumor samples were categorized into high and low R-loop groups according to the R-loop score, and the proportions of malignant cells, immune cells, and other cell types in both groups were calculated, respectively. Interestingly, the results indicated that immune cells constituted the predominant cell type in both groups, while the proportion of malignant cells was higher in the high R-loop score group compared to the low R-loop score group, supporting that the R-loop score might be correlated with the malignancy of HCC (**Figure 3A**). Furthermore, we assessed the expression of immune evasion molecules in the high and low R-loop score groups. As shown in **Figure 3B**, it was evident that genes related to major histocompatibility complex (MHC) and tumor-associated antigens (TAA) exhibited lower expression in the high R-loop score group. In addition, utilizing single-sample gene set enrichment analysis (GSEA), we found that the immunogenic cell death (ICD) pathway scores were markedly lower in the high R-loop score group, indicating a diminished immune response against tumors (**Figure 3C**). Collectively, these results suggested that the group with a higher R-loop score was more likely to exhibit immune evasion responses. Given that T cells, as highly heterogeneous immune cells, play a pivotal role in anti-tumor immunity, a total of 48,822 T cells were further analyzed to distinguish subpopulations using unsupervised clustering. Based on various marker genes, we identified 11 distinct T cell subpopulations, including CD8+ effector T cells, CD4+ memory T cells, natural killer T (NKT) cells, CD4+ regulatory T (Treg) cells, MAIT cells, CD4+ naive T cells, natural killer (NK) cells, CD8+ exhausted T cells, CD4+ Th1-like cells, and CD8+ memory T cells (**Figure 3D**, **Supplementary Figure S3A, B**). Notably, analysis of T cell differences between the high and low R-loop score groups revealed a remarkable increase in CD8+ exhausted T cells in the high R-loop score group, accompanied by a decrease in CD4+ naive T cells, CD4+ Th1-like cells, and NK cells, suggesting that high R-loop scores contributed to a suppressive immune microenvironment to promote tumor immune evasion (**Figures 3E, F**). Subsequently, we conducted KEGG enrichment analysis on both unregulated and downregulated differentially expressed genes (DEGs) in various T cell subpopulations with high and low R-loop scores. As shown, the upregulated genes in the high R-loop score group were significantly enriched in the TNF, IL-17, and MAPK signaling pathways, which play critical roles in the malignant progression and immune evasion of HCC (**Supplementary Figure S3C**). In addition, the downregulated DEGs significantly activated signaling pathways such as apoptosis and adherens junction (**Supplementary Figure S3D**). Moreover, to investigate the potential impact of R-loop levels on immune activation, we examined the IFN-α response in T cell subpopulations. IFN-α, a crucial immunomodulatory factor, possesses potent antitumor, antiviral, and immunoregulatory capabilities, playing an essential role in both innate and adaptive immune responses (25). The IFN-α response score in T cell subpopulations was significantly lower in the high R-loop score group compared to the low R-loop score group, suggesting that a high R-loop score suppresses immune activation (**Figure 3G**). Notably, the apoptosis scores of CD4+ Treg and CD8+ exhausted T cells were significantly higher in the low R-loop score group, which is consistent with the findings from KEGG enrichment analysis (**Figure 3H**). Taken together, the R-loop score is instrumental in assessing differences in patients’ tumor immune microenvironments, thereby providing valuable insights for selecting appropriate immunotherapy strategies.

### Differences in cell-cell interactions in high and low R-loop score groups

**Figure 4:**
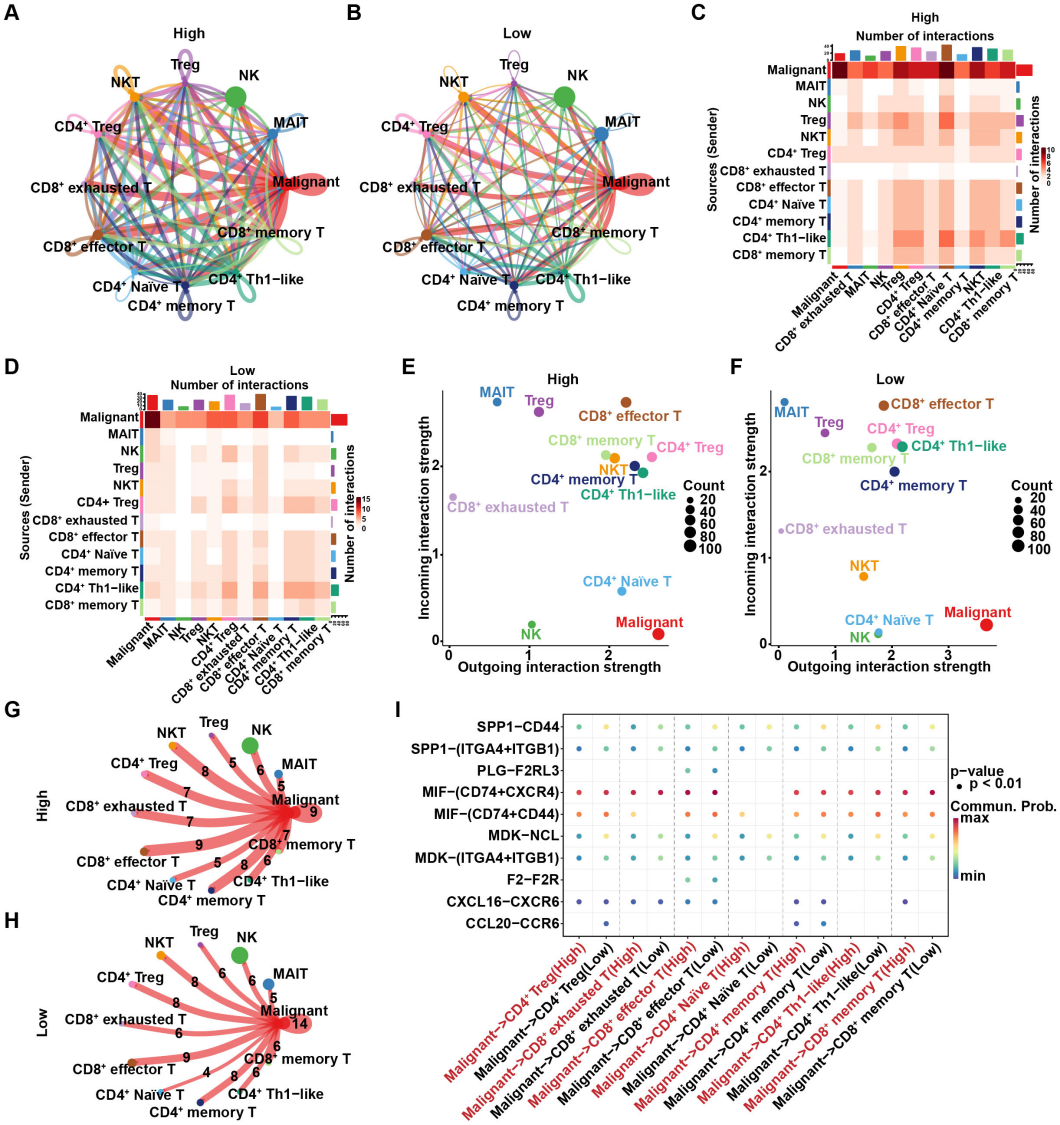
Differences in cell-cell interactions between the high and low R-loop score groups. **(A)** Number of intercellular interaction relationships between the high R-loop score group, where nodes represent cell types and arrows represent pointing relationships. **(B)** Number of intercellular interaction relationships in the low R-loop score group. **(C)** Heatmap showing the number of intercellular interactions in the high R-loop score group, with the vertical axis representing signal-giving cells and the horizontal axis representing signal-receiving cells. **(D)** Heatmap showing the number of intercellular interactions in the low R-loop score group. **(E)** Cell signaling patterns in the high R-loop score group, with the horizontal axis representing output signals and the vertical axis representing input signals. **(F)** Cell signaling patterns in the low R-loop score group. **(G)** Number of intercellular interaction relationships in the high R-loop score group, where the ligand-receptor pair count is indicated by the numbers. **(H)** Number of intercellular interaction relationships in the low R-loop score group. **(I)** Dot plot showing interactions between T cell subsets and malignant cells in the high and low R-loop score groups via ligand-receptor pairs (interaction degree is represented by color, and dot size indicates the *P*-value).

Since intercellular communication can play a crucial role in tumor immunity through various mechanisms, such as supporting immune evasion and remodeling the immune microenvironment, we hypothesized that such communication may also be involved in regulating R-loop-mediated differences in tumor immunity. Consequently, CellChat was utilized to analyze intercellular interactions among malignant cells and various T cell subpopulations. As indicated, malignant cells exhibited the highest number and intensity of interactions with other cells in both the high and low R-loop score groups, indicating strong interactions between malignant cells and other cell types and highlighting their central role in the tumor immune microenvironment. (**Figures 4A–D**). The inferred incoming and outgoing interaction strengths were elaborated in **Figures 4E, F**. Specifically, malignant cells acted as the primary signaling output cells, actively participating in intercellular interactions. Furthermore, we found that the number of intercellular interaction relationships was significantly higher in the high R-loop score group compared to the low R-loop score group (**Figures 4G, H**). Subsequently, we analyzed ligand-receptor interactions between malignant cells and T cell subtypes in the high and low R-loop score groups. As shown in **Figure 4I**, ligand-receptor-mediated interactions primarily occurred through the MIF signaling pathway (MIF-CD74+CXCR4 and MIF-CD74+CD44). Additionally, CCL20 and CCR6 were involved in interactions between malignant cells and CD4+ regulatory T cells in the low R-loop score group, whereas in the high R-loop score group, interactions were observed between malignant cells and CD8+ memory T cells involving CXCL16 and CXCR6 interaction, as well as the MIF-CD74+CD44 interaction facilitated crosstalk between malignant cells and CD8+ exhausted T cells. Altogether, these findings further elucidate the stronger intercellular communication in the high R-loop score group, which might be an important cause of the suppressive immune escape microenvironment.

### Cell trajectory analysis of malignant cells with high and low R-loop scores

**Figure 5:**
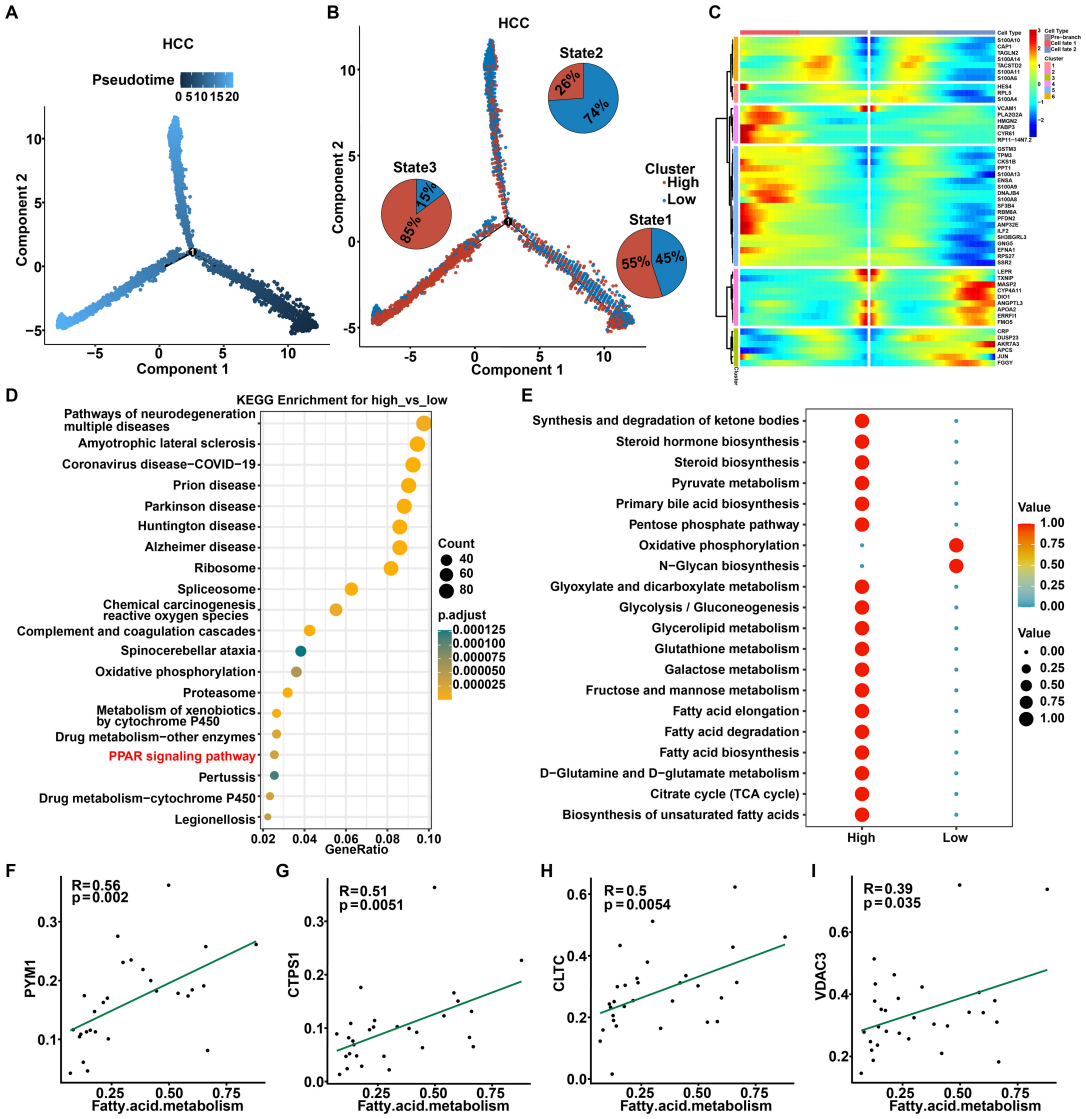
Cell trajectory analysis of malignant cells with high and low R-loop scores. **(A)** Pseudotime plot showing the trajectory of all malignant cells between the high and low R-loop score groups. **(B)** Trajectory plot indicating the locations of cells with high and low R-loop scores, with pie charts illustrating the distribution of malignant cells across different trajectory branches. **(C)** The heatmap illustrates the changes over time for the top 50 genes associated with the trajectory. **(D)** KEGG enrichment analysis of differentially expressed genes (DEGs) between the high and low R-loop score groups. **(E)** KEGG enrichment analysis of metabolic activity in malignant cells with high and low R-loop scores. **(F-I)** Pearson correlation analysis of R-loop modeling genes and the fatty acid metabolism pathway, including PYM1 **(F)**, CTPS1 **(G)**, CLTC **(H)**, and VDAC3 **(I)**.

To further explore the potential evolution of the origin, differentiation, and trajectory of malignant cells, we first utilized the Monocle2 R package to reconstruct the pseudo-time trajectory of malignant cells. As shown, at a critical branching point (Branch 1), all cells diverged into two distinct differentiation trajectories (**Figure 5A**). Interestingly, integrating the R-loop scores revealed an increase in malignant cells with low R-loop score during the transition from state 1 to state 2, and conversely, the gradual enrichment of the malignant tumor cells with high R-loop score during the transition from state 1 to state 3, highlighting that R-loop score affected the cell-state transition trajectory of malignant subpopulations. (**Figure 5B**, **Supplementary Figure S4A**). In light of this, heatmaps illustrating the molecular dynamics indicated that the alterations of the top 50 trajectory-associated genes revealed that, along with the fate 3 branch, cluster 2 genes activated were primarily enriched in the peroxisome proliferator-activated receptor (PPAR) signaling pathway (**Figure 5C**, **Supplementary Figure S4B**). Besides, KEGG analysis of differential genes between the high and low R-loop score groups further confirmed significant involvement of the PPAR signaling pathway (**Figure 5D**). PPARs, as a class of nuclear receptors abundant in adipose tissue, play a pivotal role in adipocyte differentiation and fatty acid metabolism (26). As expected, the analysis of cellular metabolism showed that fatty acid metabolic pathways were remarkably activated in malignant cells with high R-loop scores (**Figure 5E**), with key fatty acid metabolism-related genes, such as SCD and ACSL1, notably affected (**Supplementary Figure S4C**). Subsequently, we determined the correlation between 165 R-loop score modeling genes and the fatty acid metabolism pathway. Importantly, the expression of PYM1, CTPS1, CLTC, VDAC3, SRPK1, and NOSIP was positively correlated with fatty acid metabolism (**Figures 5F–I**, **Supplementary Figures S4D, E**), while the expression of FANCG, YWHAZ, and SLC25A6 showed significant negative correlations with this signaling pathway (**Supplementary Figures S4F-H**). Collectively, it is reasonable to speculate that the fatty acid metabolism pathway may play a pivotal role in mediating the regulatory effects of R-loops.

### Construct and identify the prognostic R-loop-associated gene signature using bulk RNA-seq dataset

**Figure 6:**
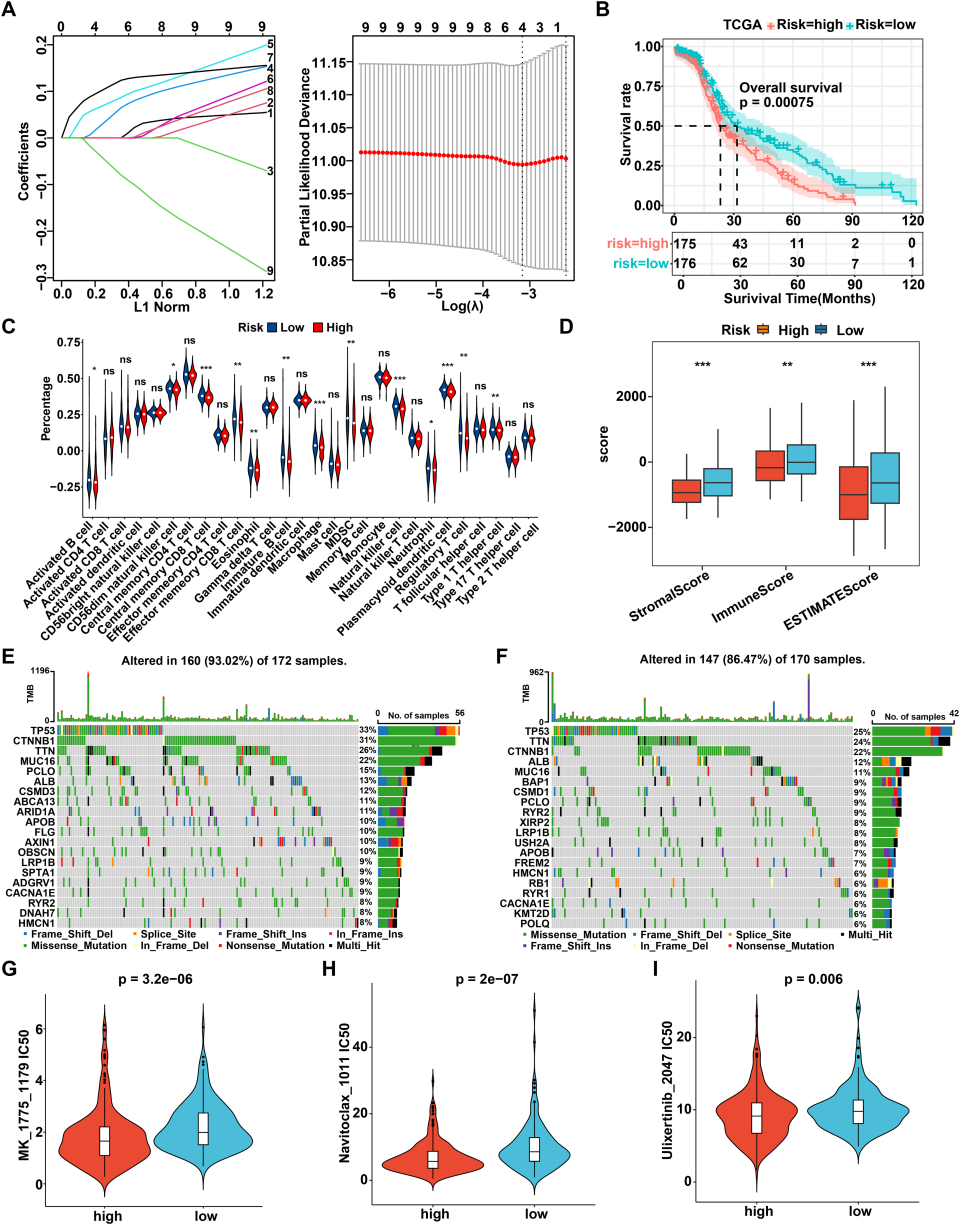
Construct and identify the prognostic R-loop-associated gene signature. **(A)** The prognostic model was constructed using eight key genes identified through Lasso analysis. **(B)** The Kaplan-Meier curve illustrates the survival differences between the high- and low-risk groups in the TCGA database according to this prognostic model (log-rank test). **(C)** Comparison of immune cell infiltration between high- and low-risk groups (Wilcoxon rank-sum test). **(D)** Comparison of tumor microenvironment scores between high- and low-risk groups (Wilcoxon rank-sum test). **(E)** Mutational landscape of patients in the high-risk group. **(F)** Mutational landscape of patients in the low-risk group. **(G-I)** Comparison of IC50 values for MK-1775 **(G)**, Navitoclax **(H)**, and Ulixertinib **(I)** between high- and low-risk groups (Wilcoxon rank-sum test). **P < 0.01; ***P < 0.001, *P* < 0.05 was considered statistically significant.

Next, utilizing the TCGA dataset as a training cohort and based on the candidate regulator genes mentioned above, eight pivotal genes (PYM1, CLTC, VDAC3, SRPK1, NOSIP, FANCG, YWHAZ, and SLC25A6) were ultimately identified by LASSO regression and multivariate Cox regression to construct a prognostic signature (**Figure 6A**). The 8-gene model formula was as follows: Risk score = (PYM1 exp) * 0.1635 + (CLTC exp) * -0.1623 + (VDAC3 exp) * 0.1596 + (SRPK1 exp) * 0.1631 + (NOSIP exp) * 0.0929 + (FANCG exp) * -0.0018 + (YWHAZ exp) * 0.0802 + (SLC25A6 exp) * -0.3306. Based on the median value of risk score, HCC patients from the TCGA, ICGC, or GSE76427 dataset were categorized into high- and low-risk groups. The survival curve indicated a poorer prognosis for patients in the high-risk group across both the training and test cohorts (**Figure 6B**, **Supplementary Figures S5A, B**). Moreover, we evaluated the prognostic value of R-loop scores using a univariate Cox model, revealing that patients in the high-risk group had significantly worse prognosis across all three cohorts compared to those in the low-risk group (**Supplementary Figure S5C**). To further investigate the relationship between risk scores and the tumor immune microenvironment, we analyzed the proportions of tumor-infiltrating immune subpopulations in the two groups. The results revealed significant upregulation of activated B cells, central memory CD8+ T cells, effector memory CD8+ T cells, immature B cells, NKT cells, and others in the low-risk group, which was consistent with the previous findings (**Figure 6C**). Moreover, the ImmuneScore, StromalScore, and ESTIMATEScore were higher and the TumorPurity score was lower in the low-risk group, suggesting a greater presence of immune components and a lower proportion of tumor components in their tumor immune microenvironment (**Figure 6D**, **Supplementary Figure S5D**). Analysis of immune checkpoint differences between high- and low-risk groups revealed that PDCD1LG2, HAVCR2, CSF1R, CD274, KDR, IDO1, and CD96 were expressed at higher levels in the low-risk group, suggesting that patients in this group may benefit from treatments targeting the corresponding immune checkpoint (**Supplementary Figure S5E**). Subsequently, we sought to investigate the relationship between R-loop scores and patients’ responses to immunotherapy. Nevertheless, immunotherapy datasets for HCC, including GSE215011 and GSE279750, were unsuitable for evaluating the association between R-loop levels and prognosis due to the lack of prognostic information in both datasets. Consequently, we attempted to analyze the impact of R-loop scores on patients’ responses to immunotherapy using datasets from other cancers, including breast cancer, melanoma, and urothelial cancer. Our results indicated that patients who received immunotherapy in the high-risk group had significantly poorer OS compared to those in the low-risk group in five independent cohorts, including GSE25055 (BRCA, *P* < 0.0001), GSE78220 (MM, *P* < 0.0001), GSE91061 (MM, *P* = 0.034), GSE176307 (UC, *P* = 0.01), and imvigor210 (UC, *P* = 0.00011) (**Supplementary Figure S5F**). Furthermore, patients who responded to immunotherapy had lower risk scores compared to those in the non-responder group in four datasets, such as GSE78220 (MM, *P* < 0.05), imvigor210 (UC, *P* < 0.05), GSE91061(MM, *P* > 0.05) and GSE176307 (UC, *P* > 0.05) (**Supplementary Figure S5G**). Meanwhile, immune response-related factors, antigen presentation and costimulatory molecules were downregulated in the high-risk group, while co-inhibitory molecules were upregulated, which is consistent with the findings (**Supplementary Figures S5H-K**). In conclusion, we confirm that the prognostic prediction risk model established based on RLRGs is indeed related to the immune status, which could effectively predict the prognosis and response to immunotherapy in the tumor patients.

Typically, genetic mutations can affect tumor progression and drug treatment response by modifying the molecular genetic profile of tumor cells. **Supplementary Figure S6A** illustrated a comprehensive overview of the TCGA-LIHC mutation data, indicating that the most prevalent category of variant classification was missense mutations, with single nucleotide polymorphisms being the most common type of variant type. Furthermore, it was observed that the median value of variants across each sample was 73, while also displaying the top 10 mutated genes. In detail, through merging the mutation data with the high- and low-risk groups, we analyzed the top 20 mutated genes, and the waterfall diagram was drawn, respectively. As shown, the top 20 mutated genes had a mutation frequency of 93.02% in the high-risk group and 86.47% in the low-risk group, with TP53 exhibiting the highest mutation frequency among all genes in both risk groups (**Figures 6E, F**). Finally, we further explored the differential response to 198 chemotherapeutic drugs between the high- and low-risk groups. The susceptibility to various chemotherapeutic agents differed between the two groups, among which 11 drugs were identified as potentially effective treatments for HCC patients in the high-risk group, including MK-1775, Navitoclax, Ulixertinib, AZD7762, Wee1 inhibitor, Paclitaxel, BI 2536, MG-132, AZD6738, Telomerase Inhibitor IX, and Sepantronium bromide (**Figures 6G-I**, **Supplementary Figures S6B-I**). Overall, a novel prognostic model based on 8 R loop-related genes is constructed and validated in HCC, which is robustly connected to the genomic heterogeneity and immunosuppressive status of patients, thereby providing an option for drug therapy.

### CLTC regulates lipid metabolism by affecting R-loop formation in HCC

**Figure 7:**
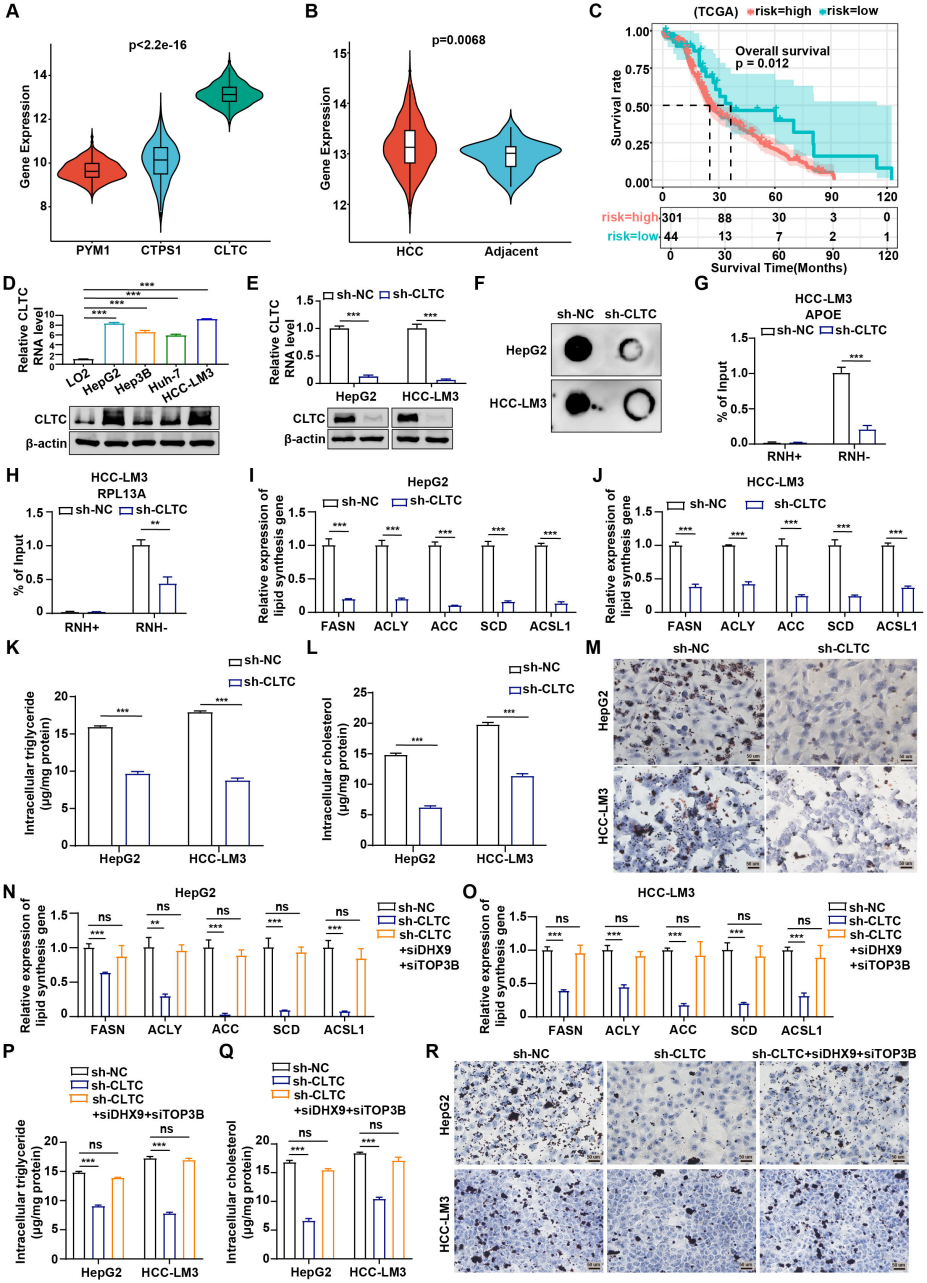
CLTC regulates lipid metabolism by affecting R-loop formation in HCC. **(A)** Differential expression of PYM1, CTPS1, and CLTC in tumor tissues from TCGA-LIHC (Kruskal-Wallis test). **(B)** Differences in CLTC expression between tumor and normal tissues in TCGA-LIHC (Wilcoxon rank-sum test). **(C)** Overall survival differences between patients with high and low CLTC expression in TCGA-LIHC (log-rank test). **(D)** qRT-PCR and western blot assays were used to measure the expression of mRNA and protein levels of CLTC in four different types of human HCC cell lines and normal human liver cells. **(E)** qRT-PCR and western blot analysis of CLTC in HepG2 and HCC-LM3 cells using control shRNA (NC) or CLTC-specific shRNA (sh-CLTC). **(F)** Dot blot showing differences in R-loop formation after CLTC knockdown in HepG2 and HCC-LM3 cells. **(G, H)** DRIP-qPCR was used to assess APOE **(G)** and RPL13A **(H)** expression in HCC-LM3 cells with CLTC knockdown compared to negative controls, with RNase H treatment as a control. **(I, J)** Expression of the lipogenic enzyme genes FASN, ACLY, ACC, SCD, and ACSL1 was measured by qRT-PCR in HepG2 **(I)** and HCC-LM3 **(J)** cells expressing sh-NC and sh-CLTC. GAPDH was utilized as an internal control. **(K)** Measurement of intracellular triglyceride levels in HepG2 and HCC-LM3 cells transfected with sh-CLTC. **(L)** Measurement of intracellular cholesterol levels in HepG2 and HCC-LM3 cells transfected with sh-CLTC. **(M)** Oil Red O staining was used to assess the effect of CLTC on lipid droplets in HepG2 and HCC-LM3 cells. **(N, O)** Expression of the lipogenic enzyme genes FASN, ACLY, ACC, SCD, and ACSL1 was measured by qRT-PCR in HepG2 **(N)** and HCC-LM3 **(O)** cells with DHX9 and TOP3B silencing after CLTC knockdown. GAPDH was utilized as an internal control. **(P)** Measurement of intracellular triglyceride levels in HepG2 and HCC-LM3 cells with DHX9 and TOP3B silencing after CLTC knockdown. **(Q)** Measurement of intracellular cholesterol levels in HepG2 and HCC-LM3 cells with DHX9 and TOP3B silencing after CLTC knockdown. **(R)** Oil Red O staining was used to assess lipid droplets in HepG2 and HCC-LM3 cells with DHX9 and TOP3B silencing after CLTC knockdown. The following notation indicates statistically significant differences: **P < 0.01; ***P < 0.001, ns, not significant. *P* < 0.05 was considered statistically significant (two-tailed t test). ns, not significant.

As previously mentioned, PYM1, CTPS1, and CLTC showed the strongest correlation with fatty acid metabolism. To further examine whether lipid metabolism was associated with RLRGs and to identify the key factor involved, we compared the mRNA levels of PYM1, CTPS1, and CLTC in HCC samples from the TCGA cohort and found that CLTC levels were significantly upregulated in clinical HCC tissues relative to the other genes (**Figure 7A**). Moreover, CLTC expression was more highly enriched in HCC tumor samples than in the corresponding normal tissues (**Figure 7B**). Of note, HCC patients with higher CLTC expression showed poorer survival outcomes compared to those with lower CLTC expression (**Figure 7C**). Collectively, these data suggest that CLTC, as the key RLRG, is associated with a worse clinical prognosis of HCC patients. Consequently, we selected CLTC for the functional experiment validation. Firstly, we measured the expression levels of CLTC in HCC cell lines (HepG2, Hep3B, Huh-7, and HCC-LM3) and a normal human liver cell line (LO2). RT-qPCR and western blot analysis exhibited a remarkable upregulation of CLTC in HCC cell lines compared to the LO2 cells (**Figure 7D**). Before loss-of-function assays, the knockdown efficiency of CLTC was assessed by RT-qPCR and western blot assays in HCC-LM3 and HepG2 cells. The results showed that CLTC expression was observably down-regulated in sh-CLTC-transfected cells compared to the negative control (**Figure 7E**). We next examined the effect of CLTC deficiency on R-loop formation and found that CLTC knockdown significantly reduced total R-loop formation in HCC cells, as determined by the S9.6 immuno-dot blot assay (**Figure 7F**). The APOE, RPL13A, and EGR1 loci, which are known to be involved in R-loop structures, were found to pause RNA Pol II transcription by forming R-loop structures (27). Meanwhile, the R-loop complex was sensitive to RNase H, which hydrolyzes RNA in RNA/DNA hybrids. We further conducted RNA-DNA immunoprecipitation and qPCR (DRIP-qPCR) analysis in HCC-LM3 and HepG2 cells. We noted a depletion of R-loop formation in sh-CLTC-transfected cells. Importantly, when the samples were treated with RNase H, the levels of R-loops dramatically decreased, confirming that the signal detected was specific for RNA-DNA hybrids (**Figures 7G, H** and **Supplementary Figures S7A–D**), suggesting that CLTC played a key role in R-loop formation.

Next, we aimed to determine whether CLTC affected lipid metabolism in HCC cells. As reported, abnormal lipid metabolism in tumors is accompanied by dysregulation of some key lipogenic enzymes, including FASN, ACLY, ACC, SCD, and ACSL1 (28). RT-qPCR results displayed that the expression of the key lipid synthesis genes was significantly down-regulated following CLTC suppression (**Figures 7I, J**). Besides, Pearson correlation analysis showed that CLTC RNA level was positively correlated with the RNA levels of the key lipid synthesis genes, including FASN (r = 0.17, *P* = 0.0021), ACLY (r = 0.39, *P* = 5.4e-14), ACC (r = 0.31, *P* = 2.6e-09), SCD (r = 0.58, *P* = 0.00079), and ACSL1 (r = 0.54, *P* = 0.0019) (**Supplementary Figure S7E-I**). Moreover, the levels of triglycerides and cholesterol in HCC cells with CLTC knockdown were significantly reduced compared with those in control cells (**Figures 7K, L**). The effect of CLTC on lipogenesis in HCC cells was further investigated using Oil Red O staining, which showed that CLTC inhibition decreased lipid droplet levels in HepG2 and HCC-LM3 cells (**Figure 7M**). To determine whether the regulation of lipid metabolism by CLTC was mediated through R-loop formation, we conducted a rescue experiment in HCC cells. S9.6 immuno-dot blot and DRIP-qPCR assays revealed that the silencing of DHX9 and TOP3B, synergistic suppressors of promoter-associated R-loops, reversed the reduction in R-loop levels caused by CLTC knockdown (**Supplementary Figures S7J-P**). As indicated, RT-qPCR assays demonstrated that silencing CLTC significantly downregulated the mRNA levels of lipid metabolism-related genes, which could be partially reversed by restoring R-loop levels (**Figures 7N, O**). Additionally, the restoration of R-loop partially rescued the inhibitory effect of CLTC knockdown on the levels of triglycerides and cholesterol (**Figures 7P, Q**). Oil Red O staining revealed that CLTC knockdown significantly reduced lipogenesis in HCC cells, while restoration of R-loop could partially alleviate this phenotype (**Figure 7R**). Therefore, we conclude that CLTC as a key RLRG could regulate lipid metabolism by affecting R-loop formation in HCC cells.

### CLTC promotes tumor progression in HCC

**Figure 8:**
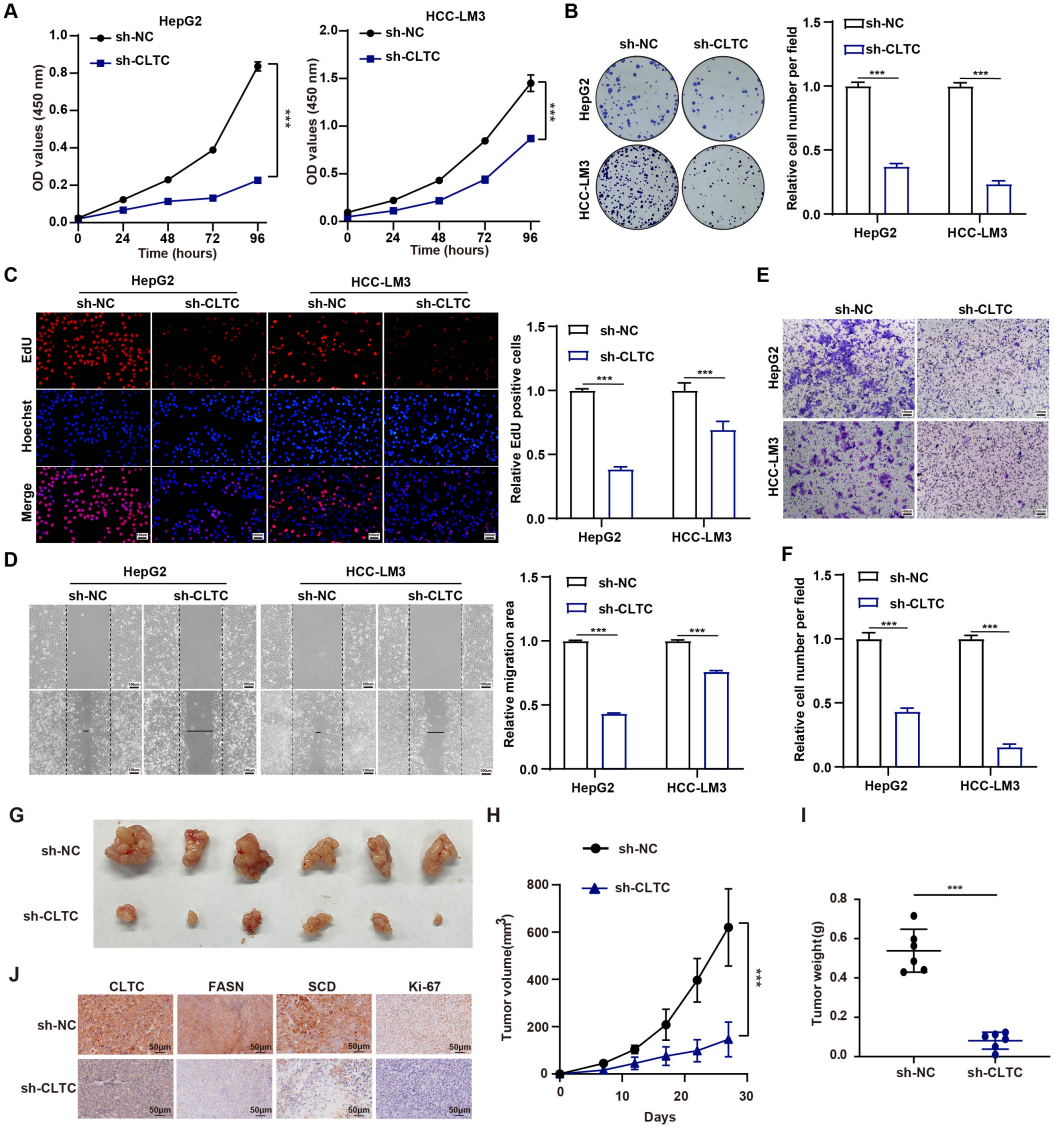
CLTC promotes tumor progression in HCC. **(A)** The CCK-8 assay was utilized to assess the proliferation capacity of HepG2 and HCC-LM3 cells expressing sh-NC and sh-CLTC. **(B)** Colony formation of HepG2 and HCC-LM3 cells was significantly reduced in the sh-CLTC group compared to the sh-NC group. **(C)** The proliferation of HepG2 and HCC-LM3 cells expressing sh-NC and sh-CLTC was evaluated using the EdU assay. **(D)** Scratch wound-healing assays were used to assess the migration of HepG2 and HCC-LM3 cells expressing sh-NC and sh-CLTC. **(E, F)** The impact of CLTC on cell migration was investigated using transwell assays in HepG2 and HCC-LM3 cells. **(G)** Representative images of the xenograft tumors derived from the indicated HepG2 cells. **(H, I)** Tumor volumes **(H)** and weights **(I)** of the xenografts originating from the indicated HepG2 cells. **(J)** Representative IHC staining showing the protein expression of CLTC, FASN, SCD, and Ki67 in the aforementioned groups. ***P < 0.001. *P* < 0.05 was considered statistically significant (two-tailed t-test).

Considering that CLTC regulates lipid metabolism, which is crucially linked to tumor progression, we therefore further explored whether CLTC could modulate the tumor progression. Consequently, we conducted a series of functional experiments to evaluate the role of CLTC in HCC progression. To identify the effect of CLTC on the cell proliferation in HCC, we performed CCK8, colony formation, and EdU assays. Firstly, CCK8 assays clearly showed that CLTC knockdown inhibited cell viability in HepG2 and HCC-LM3 cells at different time points (24, 48, 72, and 96 h) (**Figure 8A**). As shown in **Figure 8B**, CLTC suppression significantly decreased colony formation compared to the sh-NC group. Consistently, EdU assays revealed that the ratio of EdU-positive nuclei in the sh-CLTC group was lower than that of sh-NC group (**Figure 8C**). Taken together, our data suggest that CLTC knockdown could block the proliferation of HCC cells. Moreover, we further investigated the effect of CLTC on cell metastasis by adopting the wound healing and transwell assays in HCC cells. The wound healing assays illustrated that the wound width of the sh-NC group was remarkably shortened than that of the sh-CLTC group (**Figure 8D**). Similarly, the transwell assays revealed fewer migrated cells in the sh-CLTC group compared to the sh-NC group (**Figures 8E, F**), confirming that knockdown of CLTC could significantly diminish the migratory ability of HCC cells. To evaluate the effect of CLTC on HCC progression *in vivo*, a subcutaneous tumor model was established in BALB/c nude mice. In comparison to the sh-NC group, mice in the sh-CLTC group exhibited significantly lower tumor volume and weight, suggesting that CLTC knockdown effectively inhibited tumor growth (**Figures 8G–I**). Meanwhile, Immunohistochemical (IHC) analysis of xenograft tumors demonstrated that the protein levels of CLTC, along with the lipid metabolism-related genes FASN and SCD, were significantly decreased in the sh-CLTC group compared to the sh-NC group. Furthermore, a decrease in Ki-67 expression was observed in the sh-CLTC group compared to the sh-NC group, suggesting a reduced rate of cellular proliferation (**Figure 8J**). Collectively, these findings suggest that depriving CLTC can suppress the progression of HCC.

## Discussion

R-loops play a multifaceted role in gene regulation by aiding transcriptional termination, facilitating centromere function, promoting DNA methylation, and supporting histone modification (29–31). Dysregulation of R-loops is associated with increased genomic instability, a hallmark of cancer that contributes to its initiation and progression (32). Recent research indicates that a predictive model based on RLRGs is effective in assessing immune evasion, metabolic reprogramming, and prognosis in lung cancer patients, further confirming the strong link between abnormal R-loops and tumor progression (33). Notably, research has uncovered aberrant R-loops as a significant mechanism potentially contributing to drug resistance and tumor stemness in HCC (18, 19). Nonetheless, the precise role and underlying molecular mechanisms of abnormal R-loops and the related genes in HCC remain incompletely understood, underscoring the need for further investigation. In this study, R-loop scores emerged as an effective signature of tumor malignancy and chemotherapy response, offering a valuable tool for evaluating the tumor immune microenvironment, genetic mutations, and prognosis in HCC patients. Importantly, we found that CLTC, a key RLRG, regulated lipid metabolism by influencing R-loop formation and facilitated tumor progression in HCC.

Recently, limited research suggests that R-loops may be involved in regulating the replication and transcription processes of viral genomes. For instance, computational analysis of over 6,000 viral genomes indicates that R-loop-forming sequences are regulated in over 70% of dsDNA viruses, demonstrating their extensive distribution across viral genomes (34). The Yiu group demonstrates that, in the absence of BNRF1, SMC6 disrupts the formation of replication compartments by recognizing and binding to R-loop regions within the EBV genome, thereby regulating genome stability and infectious virion production (35). Of note, HCC patients commonly exhibit concurrent HBV infection, which reportedly facilitates the carcinogenesis and malignant progression through direct or indirect mechanisms (36). In this study, we constructed an R-loop scoring model using the 165 R-loop regulators that were significantly associated with HBV infection. As expected, the R-loop scores of HBV-infected samples were significantly higher than those of non-infected samples, suggesting a strong correlation between R-loop scores and HBV infection. Furthermore, the R-loop scores were upregulated in tumor samples and malignant cells and increased with clinical stage, indicating that R-loop formation might contribute to the initiation and malignant progression of HCC.

In the tumor immune microenvironment, complex intercellular communication among tumor cells, immune cells, and stromal cells influences tumor progression and treatment response (37). Certain studies indicate that R-loops may modulate the tumor immune microenvironment by influencing cellular homeostasis among different cell types. For example, a deficiency in Ten-eleven translocation (TET) enzyme leads to an increase in G-quadruplexes and R-loops, disrupting normal B cell homeostasis and promoting B cell lymphoma development, whereas therapies targeting R-loops enhance apoptosis in TET-deficient B cells (38). Notably, the treatment response and disease progression of HCC patients vary greatly among individuals due to their complex tumor immune microenvironment and high heterogeneity (39). During our analysis of immune cells in the tumor immune microenvironment, we found that CD4+ naive T cells, CD4+ Th1-like cells, and NK cells were significantly downregulated in the high R-loop score group, while CD8+ exhausted T cells were markedly increased. Decreased CD4+ naive T cells directly impact antigen-specific responses and the generation of effector T cells (40), while the decrease in CD4+ Th1-like T cells weakens the body’s immune surveillance and attack against tumors (41), and the decrease in NK cells impairs the ability to clear tumor cells (42). The increased presence of CD8+ exhausted T cells suggests impaired functionality, characterized by a reduced proliferative capacity and weakened tumor-killing capabilities (43). Besides, high R-loop scores were associated with lower levels of TAA, MHC, ICD and IFN-α response score. These changes suggest that R-loop accumulation mediates the formation of an immunosuppressive microenvironment, thereby facilitating immune evasion. In recent years, immunotherapy has emerged as a new beacon of hope for patients with advanced HCC, progressively becoming a focal point in both foundational and clinical research. Regrettably, only a minority of patients exhibit positive responses to immunotherapy (44). Therefore, we proposed that R-loops could serve as biomarkers to identify patients who might benefit more from immunotherapy. The regulatory role of R-loops in the tumor immune microenvironment observed in scRNA-seq data prompted us to further explore the relationship in bulk RNA-seq data. Immune evasion, facilitating tumor suppression or evasion of immune cell attacks through various mechanisms, was correlated with the extent of immune cell infiltration (45). We further observed that activated B cells, central memory CD8+ T cells, effector memory CD8+ T cells, immature B cells, NKT cells, exhibited lower expression in the high-risk group, suggesting suppression of anti-tumor immune function, potentially aiding tumor immune evasion. Immune checkpoint analysis indicates that patients in the low-risk group are more likely to benefit from immune checkpoint inhibitors (ICIs) treatment, highlighting the potential of R-loop scores to guide personalized ICIs therapy. In addition, TP53 had the highest mutation frequency in both high- and low-risk groups, with its inactivation or mutation altering T cell activity and recruitment, leading to dysregulation of the tumor immune microenvironment and immune escape. Since the therapeutic response of patients in different risk groups is influenced by their immune status, we aimed to identify drugs that are more effective for patients in high-risk groups to improve their prognosis. Based on our findings, we identified eleven drugs, including MK-1775 and Navitoclax, that show promising therapeutic potential for high-risk patients.

Cancer cells require substantial energy during their development to adapt to the demands of survival, growth, proliferation, invasion, and metastasis, which are supported by reprogrammed lipid metabolism (46). Abnormal lipid metabolism, in particular, contributes to malignant progression and drug resistance in HCC, as the liver serves as the core metabolic organ (3). R-loops reportedly may play a role in regulating cellular lipid metabolism. The SARS-CoV-2 spike protein increases R-loop formation on MSR1 mRNA, leading to inhibition of macrophage lipid uptake and thereby alleviating atherosclerosis (47). Cell trajectory analysis of our data revealed that the expression of genes related to the PPAR pathway became progressively stronger as the percentage of malignant cells with high R-loop levels increased. Meanwhile, as PPAR signaling plays an important role in adipocyte differentiation and fatty acid metabolism, we further observed that malignant cells in the high R-loop score group significantly activated fatty acid metabolism-related pathways. Subsequently, our prognostic model, established using eight RLRGs significantly associated with lipid metabolism, revealed markedly lower survival rates in the high-risk group. To the best of our knowledge, this study is the first to demonstrate a link between R-loops and lipid metabolism in tumors, and further investigation is warranted to clarify the underlying molecular mechanisms.

Clathrin, a multimeric protein complex composed of three CLTC and three clathrin light chains, plays a critical role in mitotic progression and membrane trafficking (48). CLTC has been reported to promote the development and progression of a wide range of tumors and is associated with poor prognosis. For example, CLTC facilitates the oncogenesis and progression in osteosarcoma by activating the TGF-beta and AKT/mTOR signaling pathways (49). CLTC, which is upregulated in tumor tissues, promotes tumorigenesis by altering the cellular response to TGF-β and is considered a potential biomarker for assessing prognosis and treatment efficacy in HCC (50). In this study, by integrating single-cell RNA-seq and bulk RNA-seq data with experimental validation, we proposed a novel perspective that CLTC could regulate lipid metabolism through its influence on R-loop formation, and promote the proliferation and migration of HCC cells, thus providing novel insights into targeting R-loops for HCC treatment.

## Conclusions

In conclusion, at single-cell resolution, we investigated the strong association of R-loop levels with malignancy and HBV infection of HCC and observed that elevated R-loop formation disrupts the balance of T cells and immune escape molecules, thereby creating an immunosuppressive microenvironment that promotes tumor progression. In addition, a novel prognostic model based on eight RLRGs was constructed and validated to effectively assess the tumor immune microenvironment, drug response, and prognosis in HCC patients. Notably, targeting CLTC, a critical RLRG, can effectively inhibit R-loop-mediated lipid metabolic reprogramming, and suppress the proliferation and migration of HCC, which suggests that R-loops could serve as potential therapeutic targets and prognostic biomarkers for HCC.

## Data Availability

The original contributions presented in the study are included in the article/**Supplementary Material**. Further inquiries can be directed to the corresponding authors.
