# Supplementary material for: Integrated single-cell and bulk transcriptome analysis of R-loop score-based signature with regard to immune microenvironment, lipid metabolism and prognosis in HCC
Source: Front Immunol. 2025 Jan 9;15:1487372. doi: 10.3389/fimmu.2024.1487372 (PMC11754264; doi:10.3389/fimmu.2024.1487372)
Supplement: Supplementary file 2 [file Table2.docx]

**Supplementary material and methods**

**Copy number variation** **(CNV) analysis**

Based on single-cell gene expression and chromosome sorting data, we used the inferCNV v1.14.0 package in R to differentiate malignant epithelial cells (mECs) from non-malignant epithelial cells (ECs). InferCNV analysis was performed with the following settings: cutoff = 0.1, cluster_by_groups = TRUE, tumor_subcluster_partition_method = “random_trees,” and hidden Markov model (HMM) = TRUE. To minimize false-positive calls in CNV inference, we applied the default Bayesian latent mixture (BLM) model to determine the posterior probability of changes in each cell, using a default threshold of 0.5. The k-means algorithm was then employed to perform hierarchical clustering of CNV scores for all genes in ECs and reference cells (plasma B cells). Cells with CNV scores greater than the 95th percentile value of reference cells were classified as malignant epithelial cells (mECs), while others were identified as non-malignant epithelial cells (ECs).

**Differentially expressed genes (DEGs) and gene functional enrichment analysis**

Modules associated with HBV infection were selected, resulting in a total of 165 genes. These genes were analyzed using Gene Ontology (GO) enrichment analysis, including biological process (BP), cellular component (CC), and molecular function (MF), as well as KEGG pathway enrichment analysis, employing the R package clusterProfiler. Differentially expressed genes (DEGs) in T cells between different groups were analyzed using the FindMarkers function in the Seurat package, with the selection standard set to a q-value < 0.05 and |Log2FC| > 0.25. GO enrichment analysis of the upregulated and downregulated genes in the DEGs between different groups was performed using the clusterProfiler R package. A q-value less than 0.05 was considered statistically significant for enrichment.

**Cell communication analysis**

The CellChat package (v1.5.0) was used to predict and visualize biologically significant intercellular communication. The scRNA-seq data were divided into R-loop high and R-loop low groups. For each dataset, the expression matrix and metadata were extracted from the Seurat object, and the createCellChat function was used to generate a CellChat object. After identifying highly variable genes and pathways, the computeCommunProb function was applied to infer the probability of intercellular communication. The results were presented using various visualizations provided by CellChat, such as netVisual_bubble, which displays a dot plot of signaling pathways emanating from cells. Additionally, the functions compareInteractions, netVisual_diffInteraction, and netVisual_heatmap were used to compare the relative number or intensity of interactions between different cell subpopulations in the high and low R-loop groups.

**Metabolic analysis**

Single-cell metabolic activity was quantified using scMetabolism (v0.2.1) for scRNA-seq metabolic analysis^[22]^. The scMetabolism software included 85 KEGG pathways and 82 Reactome entries, employing a visual algorithm to determine the activity score for each metabolic pathway in each cell. Genes related to fatty acid metabolism were downloaded from MsigDB, resulting in 177 genes. Differentially expressed genes (DEGs) between high and low R-loop score subgroups were analyzed using the FindMarkers function from the Seurat package, with adjusted *P*-value < 0.05 as the screening criterion.

**Cell trajectory analysis**

To further investigate the differentiation relationships of malignant cell subpopulations in the R-loop high and low subgroups, we selected malignant cells from both subgroups for cell trajectory analysis. The analysis was conducted and visualized using Monocle 2 (v2.28.0), which employs machine learning techniques based on reverse graph embedding from scRNA-seq data. Monocle 2 utilizes the DDRTree method for dimension reduction to construct a tree structure, iteratively refining it until both the tree structure and cellular positions converge. Based on the preceding dimensionality reduction plots, Monocle 2 learns to delineate trajectories that describe how cells transition between different states.

**Immune cell infiltration analysis**

The analysis of immune cell populations, immune functions, and immune pathway activities within each sample was conducted using single-sample gene set enrichment analysis (ssGSEA), focusing on immune competence. The proportions of stromal and immune cells in each tumor sample were estimated using the Estimation of Stromal and Immune cells in Malignant Tumor tissues using Expression data (ESTIMATE) algorithm, which calculates the sum of quality expression for both cell types. Additionally, we examined the relationship between the risk score and the expression levels of immune checkpoint-related genes.

**Gene Mutation Analysis**

Differences in gene mutations between the risk groups were analyzed using the Maftools R package, based on single nucleotide polymorphism (SNP) mutation site data for HCC.

**Drug sensitivity analysis**

The half-maximal inhibitory concentration (IC50) values of 198 commonly used chemotherapeutic drugs from the GDSC2 database (https://www.cancerrxgene.org) were obtained using the R package “oncoPredict.” The rank-sum test was used to compare IC50 differences between the high-risk and low-risk groups, with a p-value < 0.05 used to identify significant drugs. The correlation between IC50 values and risk scores was analyzed using Spearman's correlation (correlation coefficient < -0.2, p < 0.05) to further evaluate the potential of patient risk scores in guiding chemotherapy feasibility. Drug target genes were obtained from the Pharmacodia database (https://data.pharmacodia.com).

**CCK-8 and colony formation assays**

For the CCK-8 assay, 2×10^3 HCC-LM3 or HepG2 cells, either treated or untreated, were seeded into individual wells of a 96-well plate. At 0, 24, 48, 72, and 96 hours after seeding, 10 μl of CCK-8 reagent (Yeasen, Shanghai, China) was added to each well. After two hours of incubation, absorbance was measured at 450 nm using a microplate reader (BioTek Instruments, Winooski, USA). For the colony formation assay, treated or untreated cells were seeded at a density of 500 cells per well in a 6-well plate and cultured for two weeks. Colonies were then fixed with 4% paraformaldehyde for 20 minutes and stained with 0.1% crystal violet for 20 minutes. Finally, images were captured using an optical microscope (Olympus, Tokyo, Japan), and ImageJ software was used for subsequent analysis.

**Wound healing assay**

Both treated and untreated cells were uniformly seeded in a 6-well plate. Once the cells formed a monolayer and reached full confluence, a 1 mL pipette tip was used to create a scratch, followed by phosphate-buffered saline (PBS) washing to remove debris and detached cells. Subsequently, cells were cultured in serum-free medium. Images were captured at 0 and 24 hours using an optical microscope (Olympus, Tokyo, Japan) and quantified using ImageJ software. The migration rate was calculated as follows: Migration rate = (initial wound area - 24-hour wound area) / initial wound area.

**Transwell assay**

The migratory capacity of HCC cells was evaluated using a transwell assay. A total of 200 μl of serum-free DMEM was used to suspend treated or untreated cells (3 × 10^5 HCC-LM3 cells or 4 × 10^3 HepG2 cells), which were then added to the upper chamber of each Corning Costar insert (Corning, NY, USA). The lower chamber of the 24-well culture plate contained 500 μl of DMEM supplemented with 20% fetal bovine serum and 1% penicillin-streptomycin. After 24 hours of incubation at 37 °C, cells on the lower surface of the membrane were fixed with 4% paraformaldehyde for 20 minutes and stained with 0.1% crystal violet for 20 minutes. Images were subsequently captured using an Olympus optical microscope (Tokyo, Japan) and analyzed using ImageJ software.

**Quantitative real‐time polymerase chain reaction (qRT-PCR)**

Total RNA was extracted using TRIzol reagent (Yeasen, Shanghai, China) according to the manufacturer's instructions, and cDNA was synthesized using a cDNA synthesis kit (Yeasen, Shanghai, China). qRT-PCR was then performed using the SYBR Green PCR kit (Yeasen, Shanghai, China) according to the manufacturer's guidelines with the CFX Connect Real-Time PCR Detection System (Bio-Rad, CA, USA). GAPDH mRNA was used as an internal control to quantify gene expression levels using the 2−ΔΔCT method. The sequences of gene-specific primers are provided in Table S1.

**Western Blot Assay**

Total proteins from the cell lines were extracted using RIPA lysis buffer containing phenylmethylsulfonyl fluoride (PMSF) and phosphatase inhibitors (Solarbio, Beijing, China). The protein samples were denatured by boiling at 100°C for 10 minutes with 5× SDS loading buffer. Equal quantities of these protein samples were subjected to electrophoresis on an SDS-polyacrylamide gel (SDS/PAGE), followed by transfer to a PVDF membrane (Millipore, Massachusetts, USA). Non-specific binding was minimized by blocking the membranes with 8% non-fat milk for 2 hours. The membranes were subsequently incubated overnight at 4°C with primary antibodies against CLTC (1:5000; Proteintech, 26523-1-AP, China) and β-actin (1:12,000; Affinity, AF7018, China). Afterward, the membranes were incubated with HRP-conjugated secondary antibodies for 1 hour at 37°C. Finally, protein bands were visualized using ECL western blotting reagents (Yeasen, Shanghai, China) and an imaging system (Bio-Rad, USA).

**5-Ethynyl-20-deoxyuridine (EdU) assay**

The EdU assay was conducted using the Cell-Light EdU Apollo488 In Vitro Imaging Kit (Beyotime, Shanghai, China). All cell groups were cultured in 50 μmol/L EdU for 2 hours at 37 °C, followed by staining according to the manufacturer's protocol. Stained cells were then observed under a fluorescence microscope, and proliferation levels were assessed as the ratio of EdU-positive cells to total cells.

**Oil Red O staining**

Both treated and untreated cells were uniformly seeded in a 6-well plate. After overnight incubation, the cells were washed twice with PBS and fixed with 4% paraformaldehyde for 20 minutes. Oil Red O staining was performed according to the manufacturer's instructions (Solarbio, Beijing, China). Stained cells were observed under a microscope and photographed.

**Plasmid construction and production of stable cell lines.**

The sh-CLTC sequence was synthesized by TSINGKE Biological Technology (Beijing, China) and cloned into the pLKO.1 vector (Promega, USA). The plasmid constructs were validated by Sanger sequencing. Plasmid transfection was performed using PEI (Yeasen, Shanghai, China) according to the manufacturer's instructions. The sequence of CLTC shRNA was as follows: CGGTTGCTCTTGTTACGGATA.

**Determination of triglyceride (TG) and Total cholesterol (TC) content**

TG and TC assay kits (Solarbio, Beijing, China) were used to measure TG and total TC content in the supernatant, following the manufacturer's protocol. TG and TC levels were calculated based on a standard curve.

**Immunohistochemical (IHC) Staining**

IHC staining was performed to evaluate the protein expression of CLTC, FASN, SCD, and Ki67. Briefly, paraffin-embedded tissue sections (4 μm) were deparaffinized with xylene and rehydrated through a graded ethanol series. Antigen retrieval was then carried out by heating the slides in citrate buffer (pH 6.0). Afterward, the tissue slides were incubated with 3% hydrogen peroxide solution to block endogenous peroxidase activity and then incubated with 5% bovine serum albumin for 30 minutes to block nonspecific binding. Primary antibodies against CLTC (Proteintech, 26523-1-AP), FASN (Proteintech, 10624-2-AP), SCD (Proteintech, 28678-1-AP), and Ki67 (Proteintech, 27309-1-AP) were applied overnight at 4°C. On the following day, the slides were incubated with horseradish peroxidase (HRP)-conjugated secondary antibody for 1 hour at room temperature, followed by staining with 3,3’-diaminobenzidine (DAB, ZSGB Bio, Beijing, China).
