# Supplementary material for: Integrated single-cell and bulk transcriptome analysis of R-loop score-based signature with regard to immune microenvironment, lipid metabolism and prognosis in HCC
Source: Front Immunol. 2025 Jan 9;15:1487372. doi: 10.3389/fimmu.2024.1487372 (PMC11754264; doi:10.3389/fimmu.2024.1487372)
Supplement: Supplementary file 3 [file DataSheet1.docx]

**Supplementary figure**

**
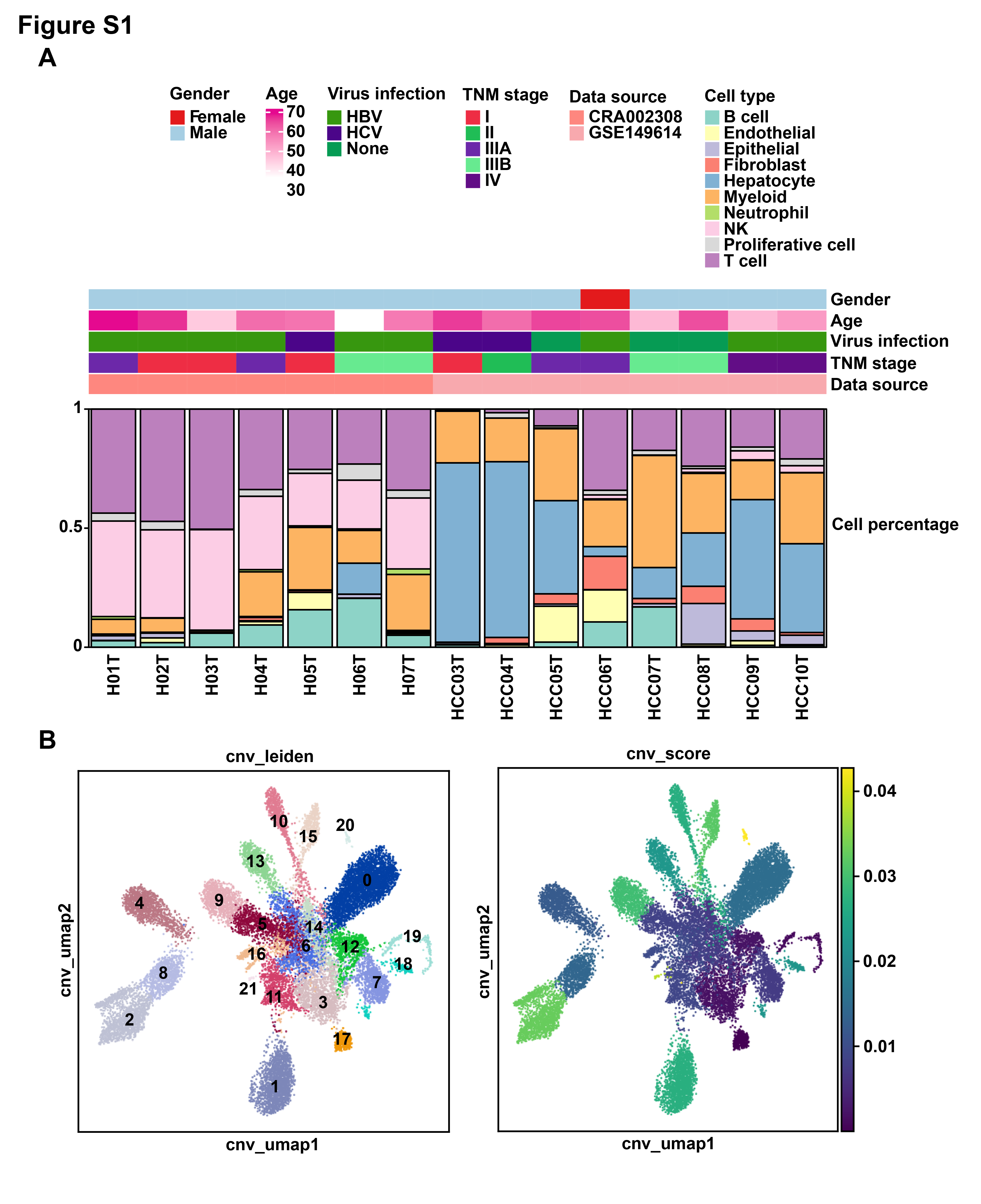
**

**Supplemental Figure 1.** Single-cell transcriptome profiles of HCC tumor and adjacent non-tumor samples. (A) Clinical features and the proportions of different cell types in each of the 15 HCC tissues that were acquired from GSE149614 and CRA002308. (B) UMAP showing the CNV status of liver and epithelial cells using plasma B cells as reference. A higher CNV score indicates a stronger likelihood of malignancy.

**
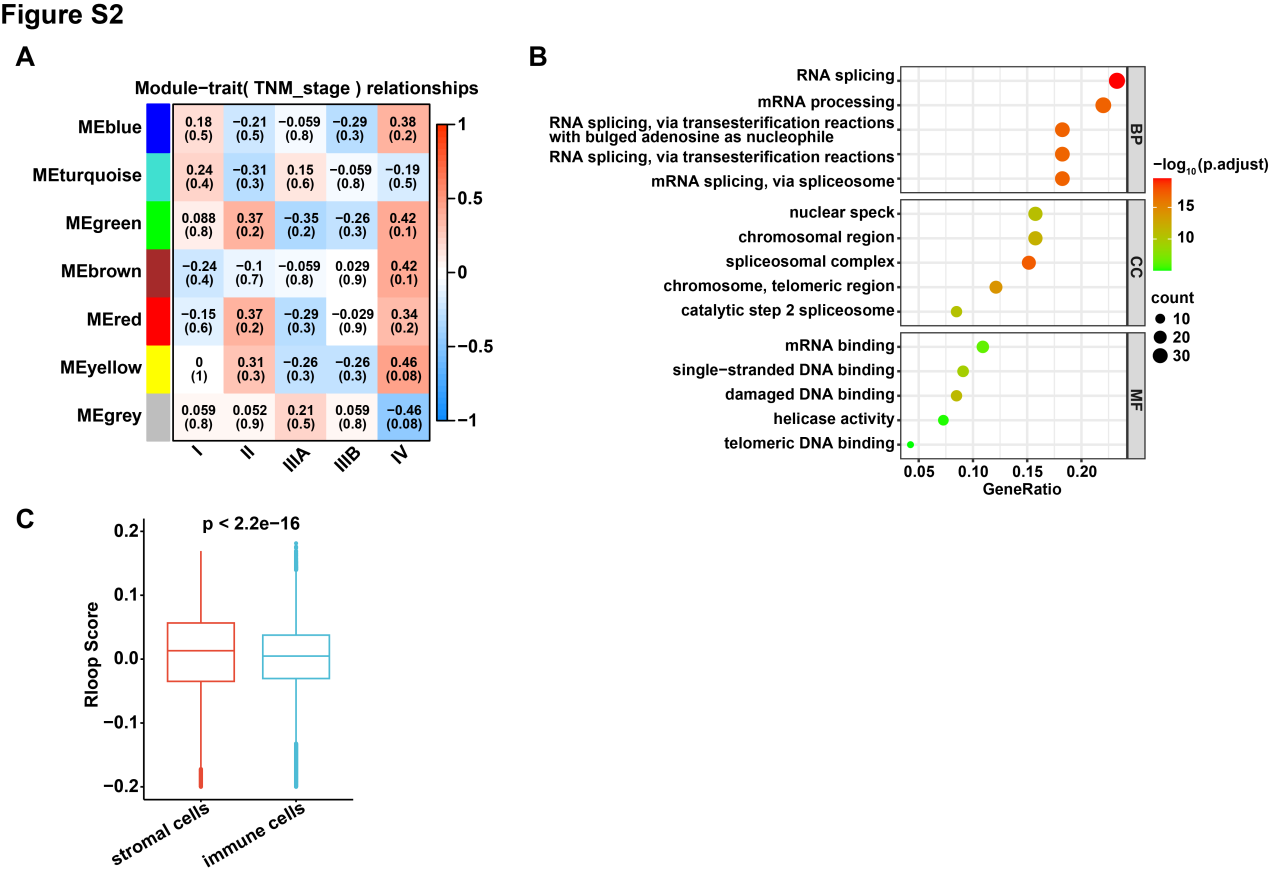
**

**Supplemental Figure 2.** The R-loop score is elevated in HCC and is associated with advanced clinical stages in HCC patients. (A) Correlation analysis of gene co-expression modules with TNM stage. (B) GO enrichment analysis of 165 genes in the blue module. (C) Comparison of R-loop scores between stromal and immune cells (Wilcoxon rank-sum test).

**
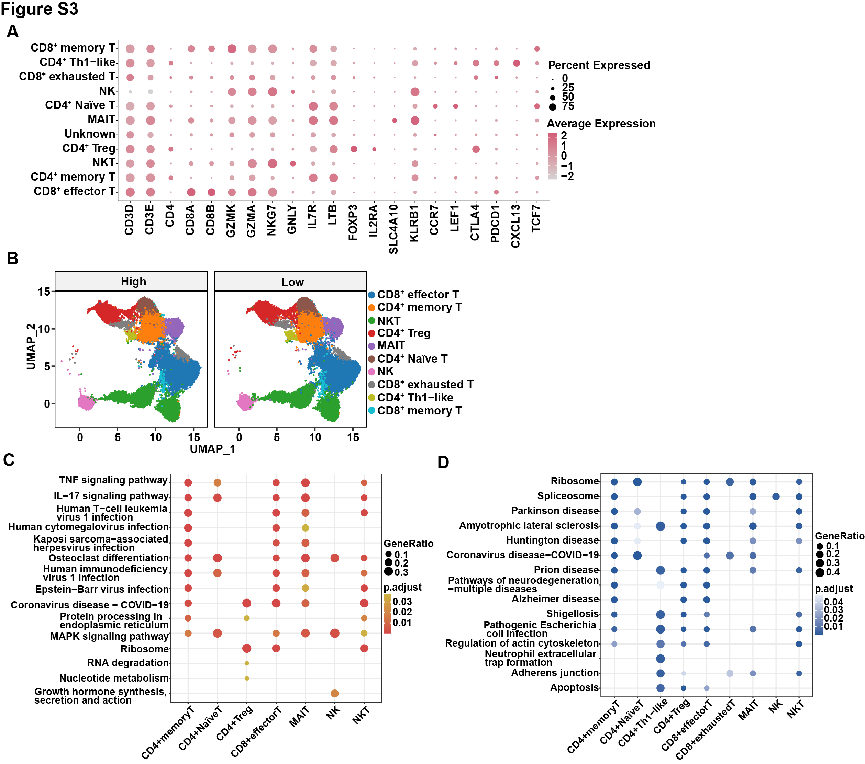
**

**Supplemental Figure 3.** R-loop score describes characteristics of tumor immune microenvironment. (A) The dot plot displays the expression of marker genes for different T-cell subpopulations. (B) Distribution comparison of cell subtypes between high and low R-loop score groups. (C) GO analysis of upregulated DEGs in T-cell subsets. (D) GO analysis of downregulated DEGs in T-cell subsets.

**
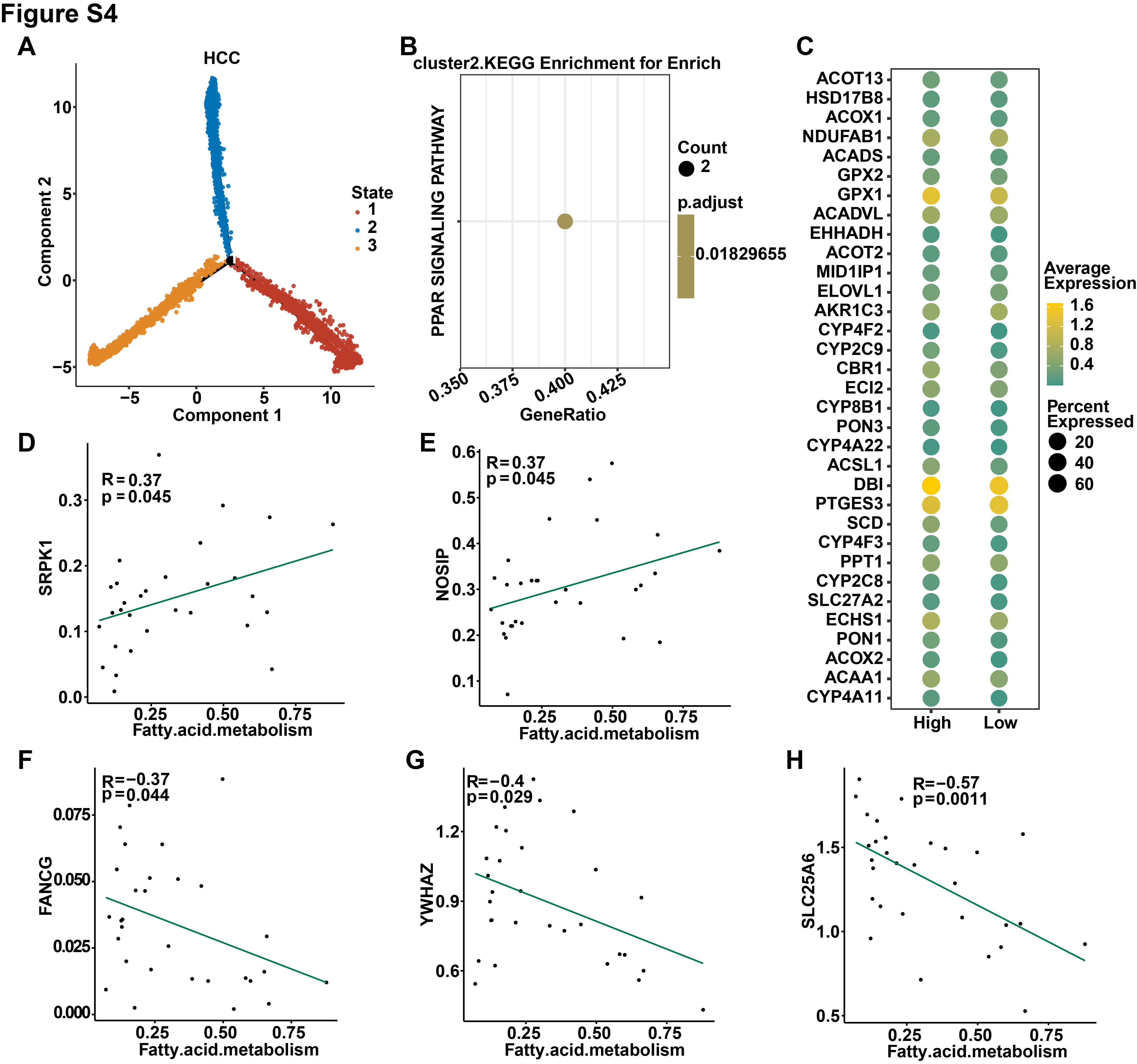
**

**Supplemental Figure 4.** Cell trajectory analysis of malignant cells with high and low R-loop scores. (A) Pseudotime plot demonstrating the bifurcation of all cells into two differentiation paths at the first major branching point. (B) KEGG enrichment analysis of genes associated with cluster 2. (C) Dot plot showing the differences in expression of fatty acid metabolism-related genes between the high and low R-loop score groups. (D-H) Pearson correlation analysis of R-loop modeling genes and the fatty acid metabolism pathway, including SRPK1 (D), NOSIP (E), FANCG (F), YWHAZ (G), and SLC25A6 (H).

**
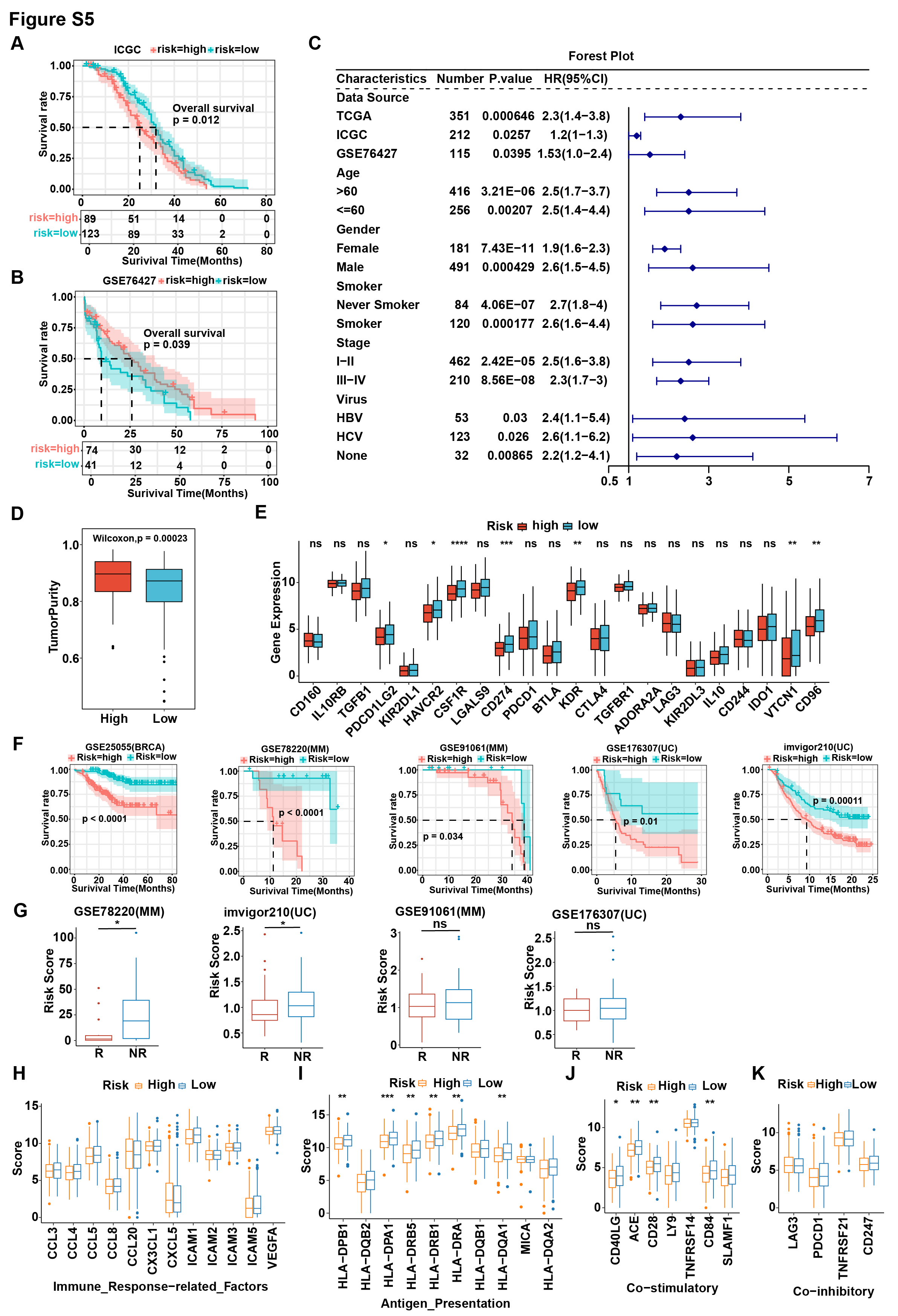
**

**Supplemental Figure 5.** Construct and identify the prognostic R-loop-associated gene signature. (A) Kaplan-Meier curve showing survival differences between the high- and low-risk groups in the ICGC database according to this prognostic model (log-rank test). (B) The Kaplan-Meier curve showing survival differences between the high- and low-risk groups in GSE76427 according to this prognostic model (log-rank test). (C) Univariate Cox analyses for the independent prognostic predictor in the training and test cohort (Wald test). (D) Comparison of TumorPurity score between high- and low-risk groups (Wilcoxon rank-sum test). (E) Analysis of immune checkpoint expression levels between high- and low-risk groups (Wilcoxon rank-sum test). (F) The Kaplan-Meier curve showing survival differences between patients who received immunotherapy in the high- and low-risk groups from GSE25055 (BRCA), GSE78220 (MM), GSE91061 (MM), GSE176307 (UC), and imvigor210 (UC) (log-rank test). (G) The difference in the R-loop score between patients who responded to immunotherapy in the high- and low-risk groups from GSE78220 (MM), imvigor210 (UC), GSE91061 (MM) and GSE176307 (UC) (Wilcoxon rank-sum test). (H-K) Comparison of immune response related factors (H), antigen presentation molecules (I), costimulatory molecules (J), and coinhibitory molecules (K) between high- and low-risk groups (Wilcoxon rank-sum test). **P*<0.05; ***P* <0.01; ****P* < 0.001; *****P*<0.0001; ns, not significant. *P* < 0.05 was considered statistically significant.

**
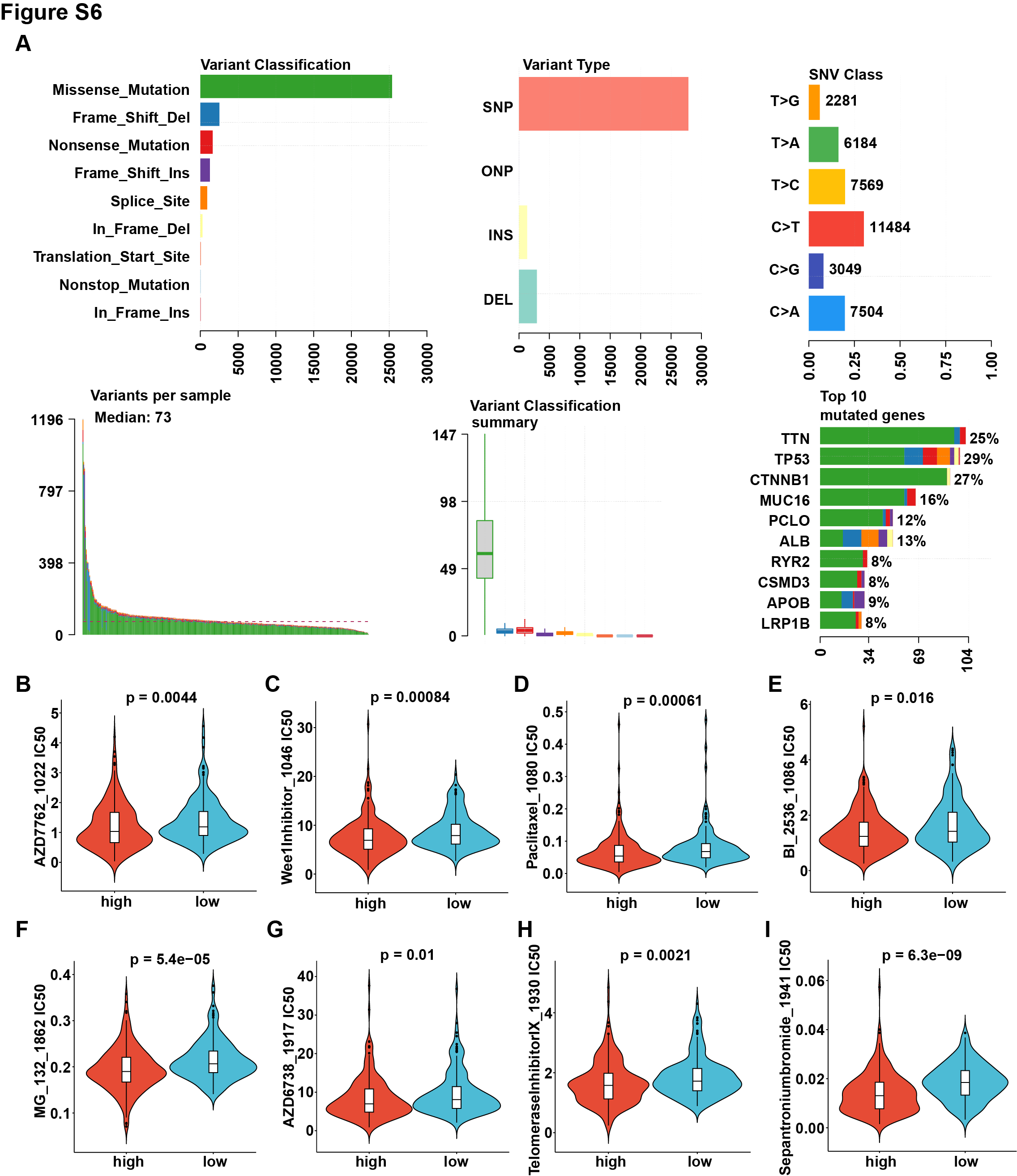
**

**Supplemental Figure 6.** Mutational landscape and comparison of IC50 values of various chemotherapeutic agents between high- and low-risk groups. (A) Overview of the mutational landscape in TCGA-LUAD patients. (B-I) Comparison of IC50 values for different chemotherapeutic drugs between the high- and low-risk groups, including AZD7762 (B), Wee1 inhibitor (C), Paclitaxel (D), BI 2536 (E), MG-132 (F), AZD6738 (G), Telomerase Inhibitor IX (H), and Sepantronium bromide (I) (Wilcoxon rank-sum test).

**
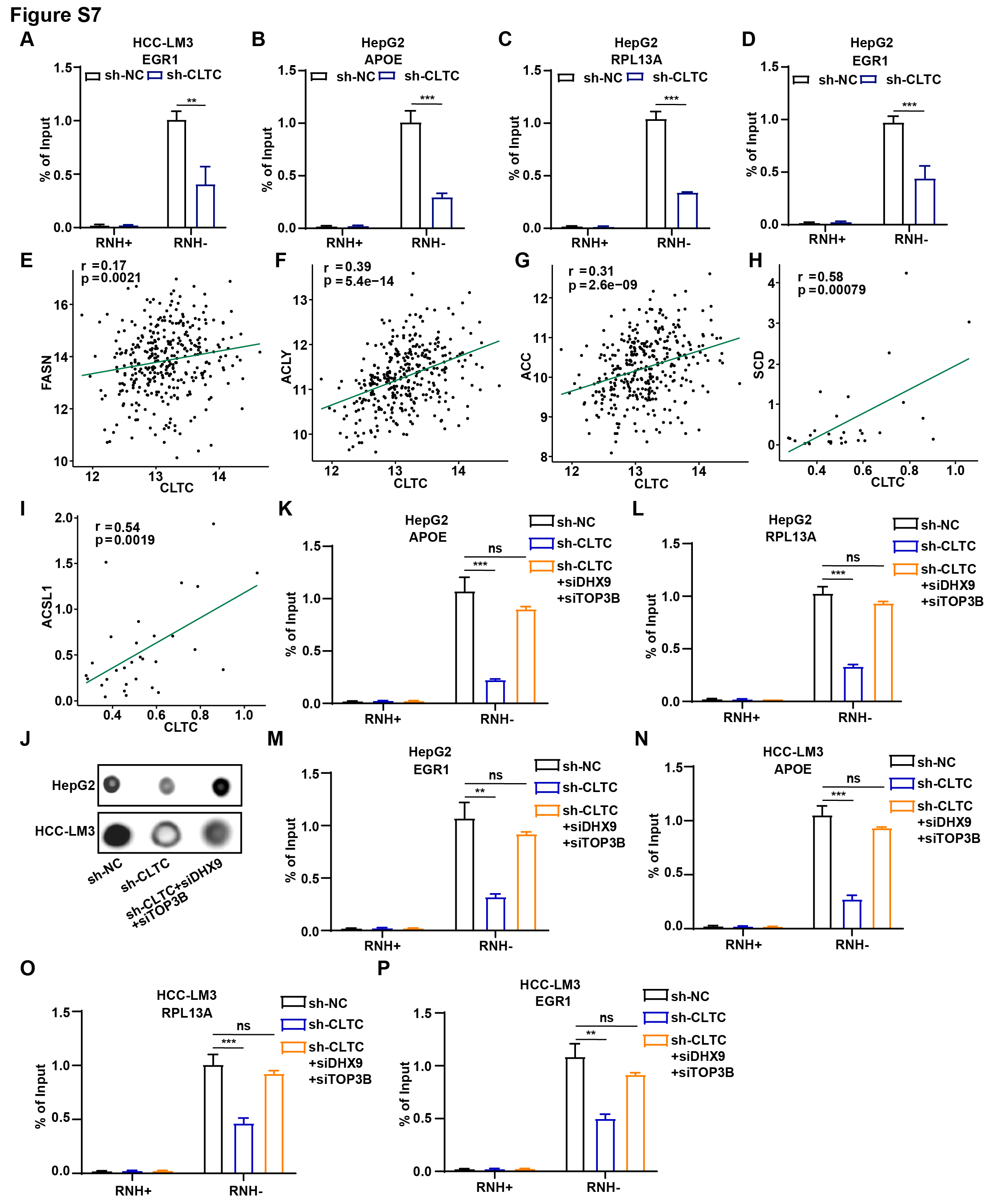
**

**Supplemental Figure 7.** CLTC could regulates lipid metabolism by affecting R-loop formation in HCC. (A) DRIP-qPCR was employed to examine EGR1 expression in HCC-LM3 cells with CLTC knockdown compared to negative controls. (B-D) DRIP-qPCR was employed to examine APOE(B), RPL13A(C), and EGR1(D) expression in HepG2 cells with CLTC knockdown compared to negative controls. (E-I) Analysis of the correlation between CLTC and lipid metabolism genes in HCC tissues, including FASN (E), ACLY (F), ACC (G), SCD (H), and ACSL1 (I). (J)Dot blot showing differences in R-loop formation in HepG2 and HCC-LM3 cells with DHX9 and TOP3B silencing after CLTC knockdown. (K-M) DRIP-qPCR was employed to examine APOE(K), RPL13A(L), and EGR1(M) expression in HepG2 cells with DHX9 and TOP3B silencing after CLTC knockdown. (N-P) DRIP-qPCR was employed to examine APOE(N), RPL13A(O), and EGR1(P) expression in HCC-LM3 cells with DHX9 and TOP3B silencing after CLTC knockdown. ***P* <0.01; ****P* < 0.001; ns, not significant. *P* < 0.05 was considered statistically significant (two-tailed t test).
